# Quantification of synthetic errors during chemical synthesis of DNA and its suppression by non-canonical nucleosides

**DOI:** 10.1038/s41598-022-16222-2

**Published:** 2022-07-15

**Authors:** Yoshiaki Masaki, Yukiko Onishi, Kohji Seio

**Affiliations:** 1 Department of Life Science and Technology, Tokyo Institute of Technology, 4259-J2-16 Nagatsuta, Midori, Yokohama, Kanagawa 226-8501 Japan; 2 JST, PRESTO, 4-1-8 Honcho, Kawaguchi, Saitama 332-0012 Japan

**Keywords:** Solid-phase synthesis, DNA, Next-generation sequencing

## Abstract

Substitutions, insertions, and deletions derived from synthetic oligonucleotides are the hurdles for the synthesis of long DNA such as genomes. We quantified these synthetic errors by next-generation sequencing and revealed that the quality of the enzymatically amplified final combined product depends on the conditions of the preceding solid phase chemical synthesis, which generates the initial pre-amplified fragments. Among all possible substitutions, the G-to-A substitution was the most prominently observed substitution followed by G-to-T, C-to-T, T-to-C, and A-to-G substitutions. The observed error rate for G-to-A substitution was influenced by capping conditions, suggesting that the capping step played a major role in the generation of G-to-A substitution. Because substitutions observed in long DNA were derived from the generation of non-canonical nucleosides during chemical synthesis, non-canonical nucleosides resistant to side reactions could be used as error-proof nucleosides. As an example of such error-proof nucleosides, we evaluated 7-deaza-2´-deoxyguanosine and 8-aza-7-deaza-2´-deoxyguanosine and showed 50-fold decrease in the error rate of G-to-A substitution when phenoxyacetic anhydride was used as capping reagents. This result is the first example that improves the quality of synthesized sequences by using non-canonical nucleosides as error-proof nucleosides. Our results would contribute to the development of highly accurate template DNA synthesis technologies.

## Introduction

Synthetic oligonucleotide is a crucial tool for diagnosis and therapeutic applications. One of the emerging applications is de novo genome synthesis, which is achieved by the assembly of a massive number of synthesized oligonucleotides^1,2^. It is believed that the capability to construct DNA sequences is doubled approximately every 3 years, allowing re-engineering of bacterial genomes^3^. For example, genomes of bacteria, such as Mycoplasma mycoides^4–6^, Salmonella typhimurium^7^, and Escherichia coli^8^, have been re-engineered by synthesis. Regarding eukaryotic genome synthesis, multiple chromosomes have been synthesized as a part of Synthetic Yeast (Sc 2.0) project^9^. One of the hurdles for these applications is the cost involved in the synthesis of de novo genomes. The cost of oligonucleotide synthesis has sharply dropped with the advances in massive parallel synthetic technology^2,10^. In contrast, the expensive and laborious sequencing validation and error correction steps are still inevitable. It is believed these steps have become a major contributor of the total cost^1^. To reduce the sequencing validation and error correction steps, improving the quality of synthesized oligonucleotides is essential.

**Figure 1:**
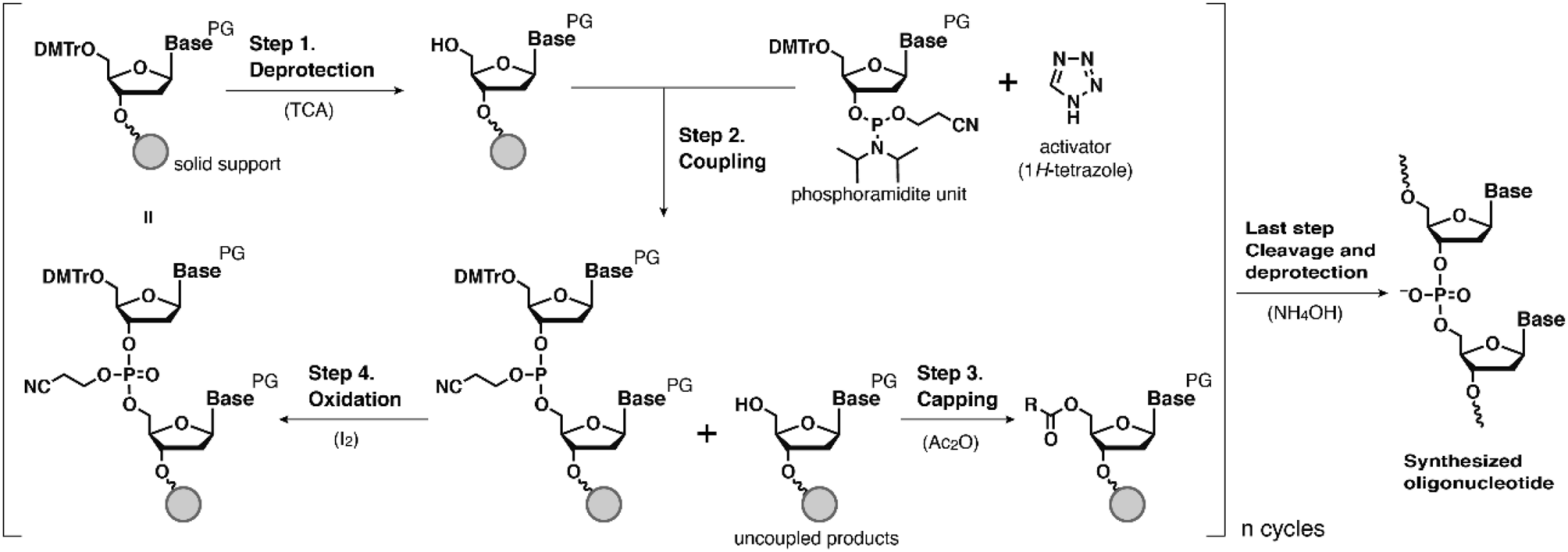
Phosphoramidite chemistry for DNA synthesis.

Current chemical synthesis of oligonucleotide was achieved by the phosphoramidite chemistry developed by Marvin H. Caruthers's group in 1981^11^. The solid-support synthesis using phosphoramidite chemistry consists of four-step synthetic cycles as shown in Fig. 1^12^. In the first step, the dimethoxytrityl (DMTr) group at the 5´-terminus nucleoside on a solid support is deprotected under acidic conditions such as 3% trichloroacetic acid (TCA) in dichloromethane. In the second step, the addition of DMTr-protected deoxynucleoside phosphoramidite and activator such as 1*H*-tetrazole to the solid support results in a coupling reaction at the 5´-hydroxy group on the solid support. In the third step, unreacted 5´-hydroxy groups are capped by an acylation reaction. In the fourth step, the oxidation of phosphite triester linkages is performed usually using 0.02 M iodine in water/pyridine/tetrahydrofuran (THF). After the synthesis is finished, the cleavage and deprotection of the protective groups on nucleobases and phosphates are performed using nucleophilic amines or ammonium hydroxide.

The efficiency of deoxynucleotide coupling is generally more than 99%. However, less than 1% of undesired products are formed in each synthetic cycle. Multiple side-products have been reported^13^. For example, overexposure of deblocking reagents leads to the formation of abasic sites due to depurination reactions^14^. Cyanoethylation at the N-3 position of thymine is caused by acrylonitrile, which is generated during the deprotection of phosphate groups^15,16^. Reagent related impurities also cause generation of side products. For example, the chloral impurity in dichloroacetic acid forms hemiacetal with the 5´-hydroxy group, which results in the insertion of chloral in the oligonucleotides^17^.

In genome synthesis, the amplification reaction by the polymerase is inevitable. Here, we have hypothesized that those side-products which are not recognized by the polymerase, can be eventually ignored. For example, cyanoethylated inosine is known to block the polymerase reaction^18^, suggesting that the prevention of base pairing can stop the amplification reaction. Strand breaks could be occurred at the abasic site, but resulting in oligonucleotides that are the fragments of the target sequence. The chloral insertion does not change the DNA sequence. By using high-fidelity polymerase and assembling reaction, these side products may be eventually ignored. Under this assumption, to increase the quality of oligonucleotide as a template DNA for genome synthesis, minimization of problematic side-products leading to substitutions, deletions, or insertions during a polymerase reaction would be critical. For example, G-to-A substitution can occur due to the formation of 2,6-diaminopurine from the guanine base^19,20^. Deprotection of the DMTr group during coupling reactions results in insertion products^21^. Insufficient capping reaction results in deletion products. Herein, we have defined substitutions, insertions, and deletions caused by the side-products during DNA chemical synthesis as synthetic errors. Importantly, these synthetic errors can be quantified using next-generation sequencing (NGS).

In this study, we quantified synthetic errors in oligonucleotides synthesized under different synthetic conditions. By using an assembling reaction for the preparation of the NGS library, only problematic side-products were evaluated in NGS. The dependency of the occurrence of synthetic errors on synthetic conditions revealed the major mechanism underlying these substitutions, which was further confirmed using non-canonical nucleosides. This result is the first example that improves the quality of synthesized sequences by using non-canonical nucleosides as error-proof nucleosides. Our result will contribute to developing a more reliable chemical synthesis of template DNA for genome synthesis.

## Results and discussion

### Evaluation of synthetic errors by next-generation sequencing

**Figure 2:**
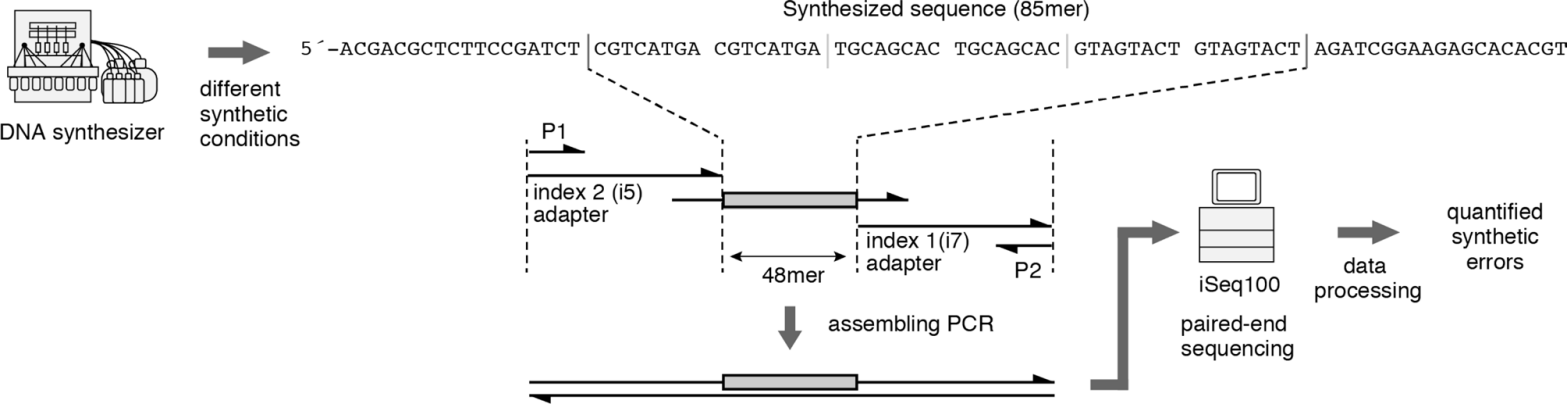
Synthetic error quantification of chemically synthesized oligonucleotides.

Chemical synthesis of DNA is known to produce various kinds of side products. Among them, we hypothesized that only those side-products, which are recognized by polymerase and cause synthetic errors (substitution, deletion, or insertion), are problematic in genome synthesis. Importantly, such synthetic errors can be quantified by next-generation sequencing. We designed a reference sequence to assess the synthetic errors encountered during chemical synthesis (Fig. 2). For the analysis of synthetic errors by next-generation sequencers, the main concern is the single nucleotide repeats. For example, in the case of a single nucleotide deletion at single-nucleotide repeats such as 5´-AA-3´, it is not possible to identify whether the 5´-A or the 3´-A has been deleted. To avoid this uncertainty, we designed a sequence that did not include any single nucleotide repeats but included all other 12 dimer combinations. The oligonucleotides with the designed sequence were synthesized by DNA synthesizer NTS-M (Nihon Techno Service Co., Ltd., Japan) under different synthetic conditions. We adopted the assembling reaction rather than the ligation reaction to prepare library constructs for the next-generation sequencer. Since synthesized DNA did not contain the 5´-primer sequence, only DNA synthesized by the polymerase was analyzed by the sequencer. The data processing of sequencing reads was slightly modified from the protocol reported earlier^22^. After merging of paired-end reads, sequence reads containing N-base call or base call with Q score less than 40 (99.99% correct base call, Q score =  − 10 log10(e), e: the probability of incorrect base call) were omitted. The alignment to the reference sequence was performed by Needleman-Wunsch aligner. In the case of chemical synthesis, insertion reaction can only occur at the 5´-end. In addition, multiple deletions or multiple insertions due to the chemical synthesis are expected to be exponentially less frequent compared to the single nucleotide deletion and insertion, and hence, were not considered (see the corresponding values in the supplementary data, Table S1). We calculated the error rates for substitution, insertion, or deletion at each sequence position and compared them between synthetic samples. The error frequencies, number of errors per kb, were also calculated.

**Figure 3:**
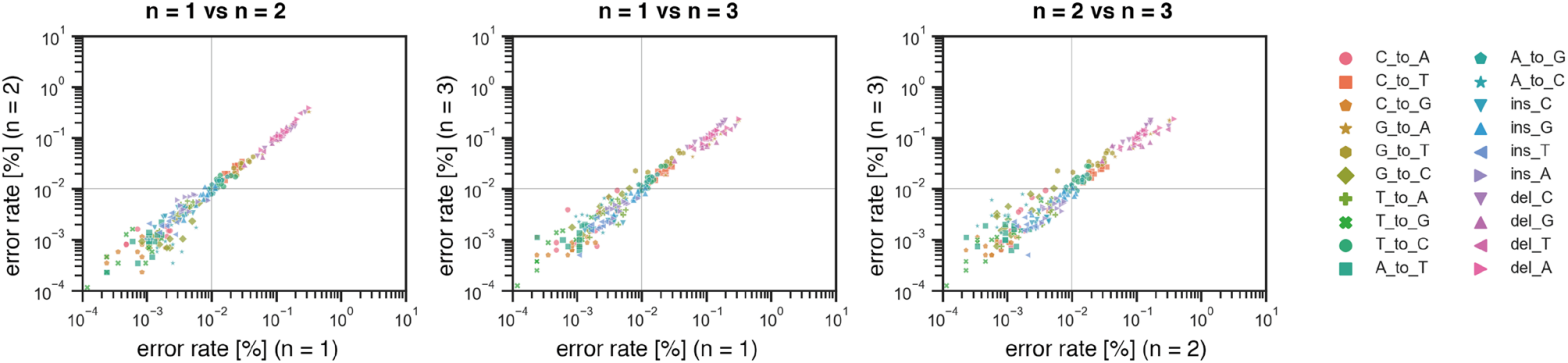
Reproducibility of observed error rates. Three oligonucleotides synthesized independently using the same synthetic conditions were compared. The synthetic conditions are 1*H*-tetrazole in anhydrous acetonitrile as an activator, acetic anhydride in THF as a capping reagent A, 10% 1-methylimidazole in 10% pyridine-THF as a capping reagent B, 0.02 M I_2_ in THF/pyridine/H_2_O (90:<1:10, v/v/v) as an oxidation reagent, and 3% trichloroacetic acid in dichloromethane (TCA) as a deblocking reagent. Q5 High-Fidelity DNA polymerase was used for assembling reaction.

First, we checked the reproductivity of sequencing results. We used 1*H*-tetrazole in anhydrous acetonitrile as an activator, acetic anhydride (Ac_2_O) in tetrahydrofuran (THF) as a capping reagent A, 10%1-methylimidazole in 10% pyridine-THF as a capping reagent B, 0.02 M I_2_ in THF/pyridine/H_2_O (90:<1:10, v/v/v) as an oxidation reagent, and 3% trichloroacetic acid in dichloromethane (TCA) as a deblocking reagent. The reactions were performed with a default setting of DNA synthesizer. For assembling reactions, Q5 High-Fidelity DNA polymerase was used. Independently synthesized three oligonucleotides were compared (Fig. 3, Fig. S1). The error rates were well reproduced down to 0.01% under our settings.

**Figure 4:**
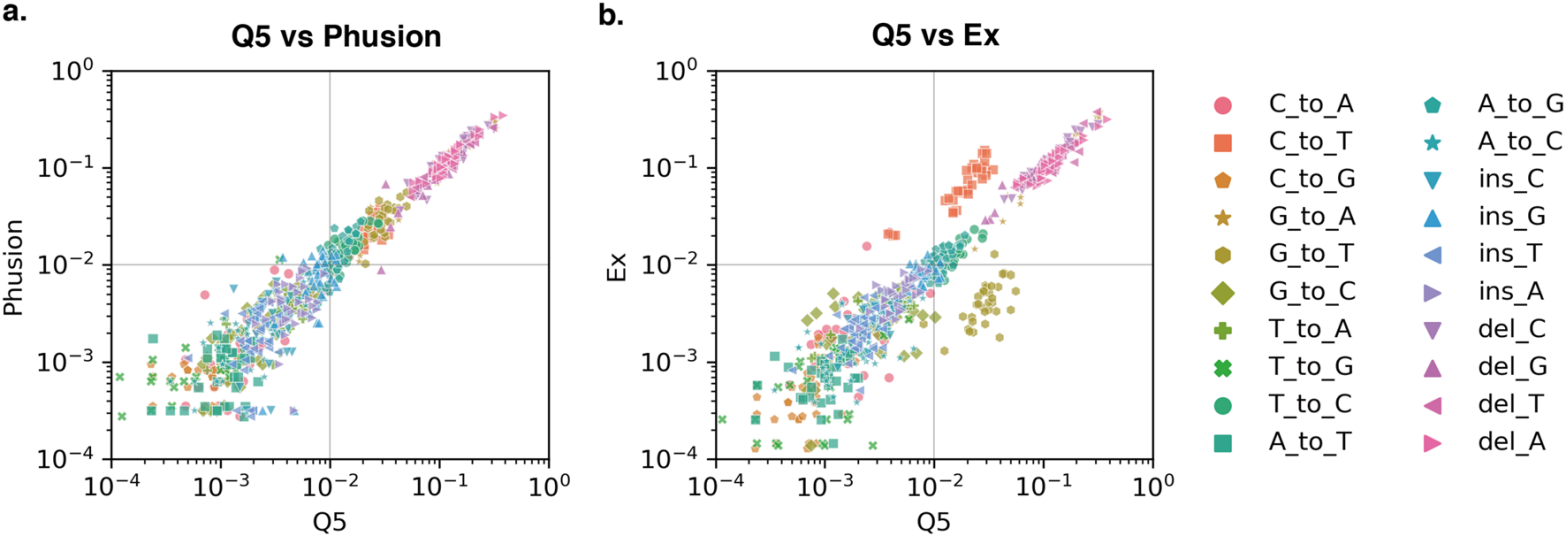
Comparison of error rates derived from assembling reactions using different polymerases. The synthesized oligonucleotide in the same batch was split into three samples and prepared NGS library by using Q5 High-Fidelity DNA polymerase (Q5), Phusion High-Fidelity DNA polymerase (Phusion), or Takara Ex Taq (Ex). (**a**) Q5 vs Phusion. (**b**) Q5 vs Ex.

We then checked the effect of DNA polymerase on the quantification of synthetic errors (Fig. 4). Assembling reaction was performed with three different polymerases, namely Q5 High-Fidelity DNA polymerase (Q5), Phusion High-Fidelity DNA polymerase (Phusion), and Takara Ex Taq (Ex), with the oligonucleotide being synthesized in the same batch. The fidelities of these polymerases reported in vender's websites were ~ 280, ~ 50, and ~ 4.5-fold higher than Taq polymerase. The observed error frequencies for Q5, Phusion, and Ex were 2.1 ± 0.13, 2.0 ± 0.30, and 2.1 ± 0.17 errors per kb, respectively. There were no significant differences, suggesting that observed substitutions, insertions, and deletions were mainly derived from the chemical synthesis process and were not due to the misincorporation by the polymerases. Interestingly, although there were no significant differences in the overall error frequencies, the observed error rate for C-to-T substitution in Ex was higher than that in Q5 and G-to-T substitution in Ex was lower than that in Q5.

In the case of C-to-T substitution, it could be a result of deoxyuridine formation, which is the deamination product of deoxycytidine. It is known that Q5 and Phusion polymerase do not efficiently read-through deoxyuridine. Thus, a higher rate of C-to-T substitution observed for Ex may stem from the recognition properties of unnatural nucleobases by each polymerase. In the case of G-to-T substitution, there are two possibilities. One possibility is the 8-oxo-deoxyguanosine formation, which is the oxidation product of deoxyguanosine. Although the read through efficiency of each enzyme for 8-oxo-deoxyguanosine has not been reported, Takara Ex Taq might be able to more efficiently avoid 8-oxo-deoxyguansine. The other possibility is the deoxyxanthosine formation, which is the deamination product of deoxyguanosine. It is known Taq polymerase preferentially incorporated deoxycytosine opposite to deoxyxanthosine, which may reduce the error rate of G-to-T substitution^23^. In genome synthesis, oligonucleotide assembling reaction should employ a polymerase with high fidelity. Hence, we decided to use Q5 High-Fidelity DNA polymerase in the following experiments.

### Synthetic errors observed under a standard synthesis condition

As a standard synthesis condition, we used 1*H*-tetrazole in anhydrous acetonitrile as an activator, Ac_2_O in THF as a capping reagent A, 10% 1-methylimidazole in 10% pyridine-THF as a capping reagent B, 0.02 M I_2_ in THF/pyridine/H_2_O (90:<1:10, v/v/v) as an oxidation reagent, and 3% trichloroacetic acid in dichloromethane (TCA) as a deblocking reagent. The reactions were performed with a default setting of DNA synthesizer NTS-M (Nihon Techno Service Co., Ltd., Japan).

**Figure 5:**
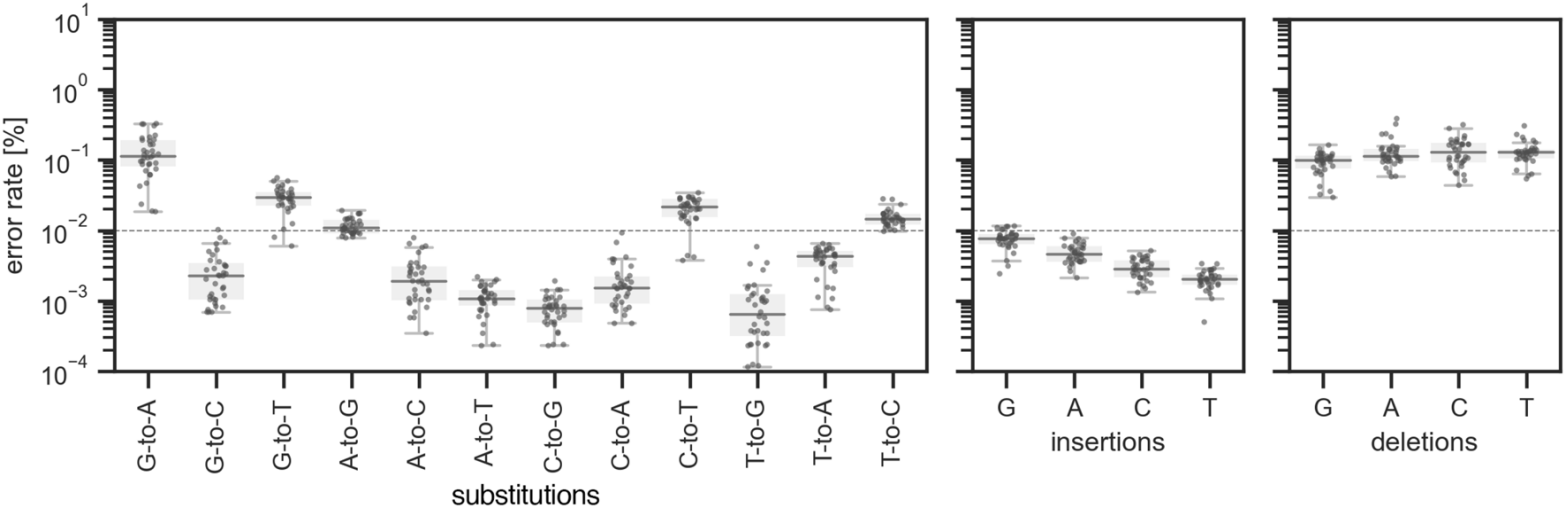
Error rates for substitutions, insertions, and deletions during standard oligonucleotide synthesis. The data were obtained from three independently synthesized oligonucleotides. The synthetic conditions are 1*H*-tetrazole in anhydrous acetonitrile as an activator, acetic anhydride in THF as a capping reagent A, 10% 1-methylimidazole in 10% pyridine-THF as a capping reagent B, 0.02 M I_2_ in THF/pyridine/H_2_O (90:<1:10, v/v/v) as an oxidation reagent, and 3% trichloroacetic acid in dichloromethane (TCA) as a deblocking reagent. Q5 High-Fidelity DNA polymerase was used for assembling reaction.

There were twelve possible patterns of substitution products (Fig. 5). Among them, the median value of error rates for G-to-A was 0.11%, which was the largest among all substitutions. The substitutions having median error rates more than 0.01% were T-to-C (0.01%), C-to-T (0.02%), A-to-G (0.01%), and G-to-T (0.03%). These substitutions could be explained by amination, deamination, or oxidation side-product^23^. It is known that 2,6-diaminopurine, which is a side product resulting from the amination of guanine base, can be recognized as adenine base by polymerases, thus resulting in G-to-A substitution. Similarly, 5-methyl cytosine resulting from amination of thymine base, uracil base resulting from deamination of cytosine base, and hypoxanthine base resulting from deamination of adenine base could be recognized as C, T, and G, respectively, by polymerases, thus resulting in T-to-C, C-to-T, and A-to-G substitutions, respectively. It is known that 8-oxo guanine base resulting from oxidation of guanine base could be recognized as T, thus resulting in G-to-T substitution. Importantly, these substitution error rates were influenced by not only the yields of side products during oligonucleotide synthesis but also the recognition ability of unnatural nucleobase by polymerases used in the assembling reaction as mentioned above. Therefore, the relative priority of substitutions that need to be addressed during genome synthesis can be estimated.

Although the products with insertions were only 0.00 ~ 0.01%, the insertion of deoxyguanosine (0.008%) was slightly higher compared to that of other nucleotides (dA: 0.005%, dC: 0.003%, and T: 0.002%). This tendency was in agreement with a previous study^21^, and was estimated to be due to the deprotection of DMTr group during the coupling reaction. About 0.1% of deletions were observed at each deoxynucleoside. It is said the coupling efficiency of standard DNA synthesis is 99%, and capping efficiency by acetic anhydride is ~ 90%. Thus, ~ 0.1% of deletions were deemed to be in a reasonable range.

### Dependency of synthetic errors on conditions of synthesis

To evaluate the influence of synthetic steps on synthetic errors, we systematically changed the reactivity of each synthetic step. For the coupling step, we synthesized the oligonucleotides using 5-benzylthio-1*H*-tetrazole (BTT, pKa 4.08)^24^ and 4,5-dicyanoimidazole (DCI, pKa 5.2)^25^ instead of 1*H*-tetrazole (Tet, pKa 4.8). For the capping step, the oligonucleotides were synthesized using a different acylation reagent (phenoxyacetic anhydride, Pac_2_O) or base (2,6-lutidine, Lut). For the oxidation step, the oligonucleotide were synthesized using 0.5 M (1S)-(+)-(10-camphorsulfonyl)-oxaziridine (CSO)^26^ instead of 0.02 M iodine. It should be noted that we used a longer oxidation reaction time (6 min) for the oxidation step in CSO than the recommended reaction time (3 min). Initially, we used recommended reaction time but the reproductivity was poor (data was not shown). To eliminate the possibility of insufficient oxidation, we used a longer oxidation reaction time. For deblocking step, the oligonucleotides were synthesized using 3% dichloroacetic acid (DCA, pKa 1.5)^14^ or 3% trichloroacetic acid (TCA, pKa 0.7) twice. The synthesized oligonucleotides were assembled with Q5 High-Fidelity DNA polymerase and applied to the next-generation sequencer.

**Figure 6:**
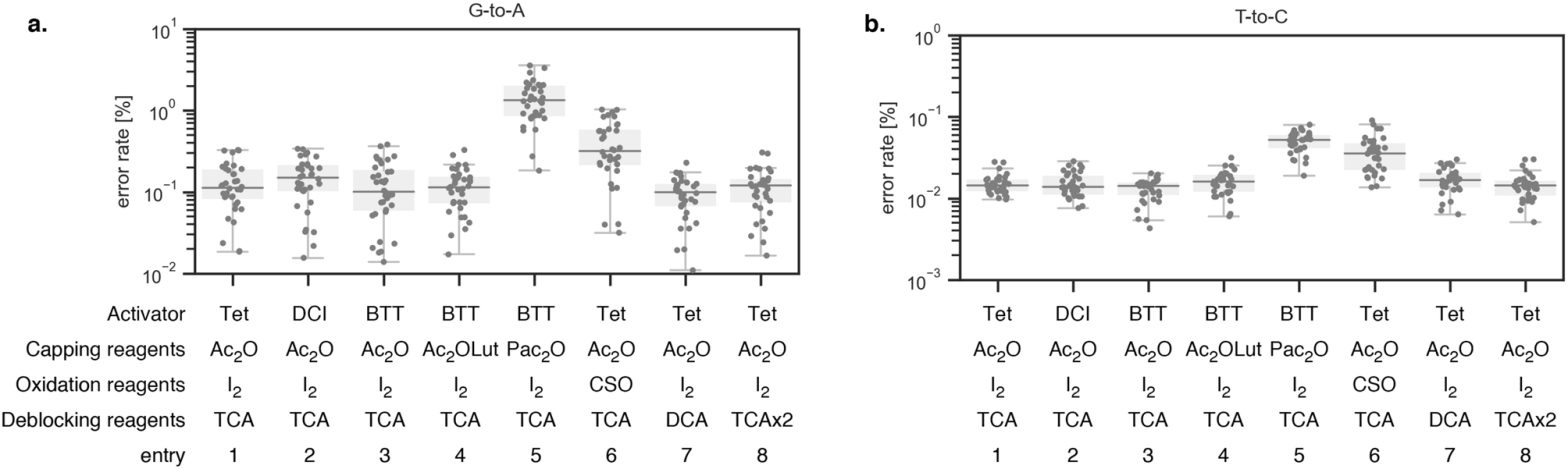
Synthetic condition dependency of error rates of G-to-A and T-to-C substitutions. The data were obtained from three independently synthesized oligonucleotides. (**a**) G-to-A substitutions. (**b**) T-to-C substitutions.

Apparent increases were observed for the error rates of G-to-A and T-to-C substitutions (Fig. 6). The boxplots for other synthetic errors are provided in the supplementary data (Figs. S2, S3, Table S2). In the case of G-to-A substitution, the median value of the error rate was increased from 0.10% (entry 3, Ac_2_O as capping reagent) to 1.33% (entry 5, Pac_2_O as capping reagent). Similarly, the median value of the T-to-C error rate was increased from 0.01% (entry 3) to 0.05% (entry 5). The G-to-A and T-to-C substitutions could be explained by the amination of guanine and thymine, which result in 2,6-diaminopurine and 5-methyl cytosine bases, respectively. Multiple mechanisms have been reported for the formation of 2,6-diaminopurine during oligonucleotide synthesis. It has been reported that nucleoside phosphoramidite can react at O-6 position of guanine base during the coupling reaction^27^. Although the resulting phosphite group is known to be cleaved by acetate anion in the next step (capping reaction)^27^, remaining phosphite group can be oxidized during the successive oxidation step. The resulting phosphate can be replaced by a nucleophilic catalyst, which can be further replaced by nucleophilic amines or ammonium hydroxide, during the cleavage and deprotection step^20^. The other mechanism involves the capping reaction^19^. The side reaction starts acylation at O-6 position of guanine base. It is not clear whether the nucleophilic catalyst is replaced; the resulting moiety would be replaced by nucleophilic amines or ammonium hydroxide at the cleavage and deprotection reaction. These mechanisms may eventually act in a similar way for the amination of thymine base to induce T-to-C substitution. In our experiments, the capping step rather than the coupling step showed larger effects, suggesting that the capping step might play a central role in G-to-A and T-to-C substitutions.

Pac_2_O is expected to show higher reactivity than Ac_2_O due to the electron-withdrawing phenoxy moiety at the alpha position. It should be noted that the median value of the error rate of deletion products under Pac_2_O conditions (0.065–0.078%) was lower than that under the Ac_2_O conditions (0.116–0.146%), suggesting higher reactivity of Pac_2_O conditions (Table S2). It is reasonable that the acylation at O-6 position of guanine base could be facilitated under Pac_2_O conditions. In addition, the acidity of phenoxyacetic acid (pKa 3.7) is lower than acetic acid (pKa 4.7), suggesting phenoxyacetoxy group could act as a better leaving group which might be also contributed to the G-to-A and T-to-C substitutions. It should be noted that the G-to-A substitution was the most prominently observed substitution among all possible substitutions even when we used Ac_2_O as capping reagents (0.12%, Table S2). Thus, it is important to develop methods to suppress G-to-A substitution to improve the quality of chemically synthesized oligonucleotide.

### Suppression of G-to-A substitution by non-canonical nucleosides

**Figure 7:**
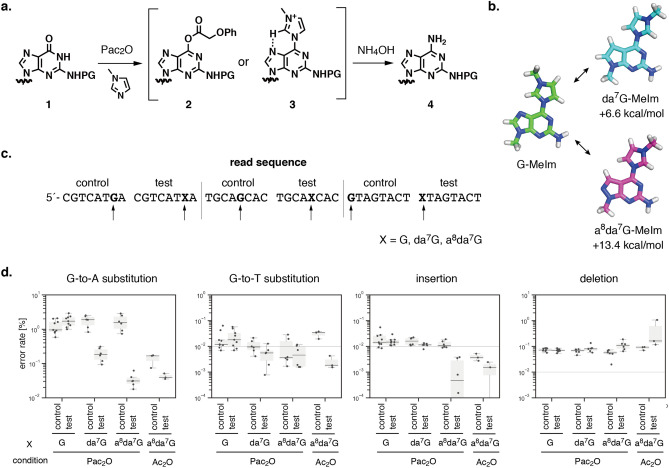
Mechanism of G-to-A substitutions. (**a**) Previously proposed mechanism of diaminopurine formation, which is slightly modified based on this study^19^. (**b**) Optimized structure of the model of intermediates. (**c**) Positions of unnatural nucleosides. (**d**) Observed error rates related to deoxyguanosine. The synthetic conditions for Pac_2_O (n = 2) are 5-benzylthio-1*H*-tetrazole in anhydrous acetonitrile as an activator, phenoxyacetic anhydride in THF as a capping reagent A, 10% 1-methylimidazole in 10% pyridine-THF as a capping reagent B, 0.02 M I_2_ in THF/pyridine/H_2_O (90:<1:10, v/v/v) as an oxidation reagent, and 3% trichloroacetic acid in dichloromethane (TCA) as a deblocking reagent. The synthetic conditions for Ac_2_O (n = 1) are 1*H*-tetrazole in anhydrous acetonitrile as an activator, acetic anhydride in THF as a capping reagent A, 10% 1-methylimidazole in 10% pyridine-THF as a capping reagent B, 0.02 M I_2_ in THF/pyridine/H_2_O (90:<1:10, v/v/v) as an oxidation reagent, and 3% trichloroacetic acid in dichloromethane (TCA) as a deblocking reagent. Q5 High-Fidelity DNA polymerase was used for assembling reaction.

Substitutions were expected to be derived from the generation of non-canonical nucleosides. In other words, non-canonical nucleosides which can be recognized by polymerase could be used in the template DNA. If non-canonical nucleosides are resistant to the side-reactions, they can be expected to suppress the synthetic errors. As an example of such error-proof nucleosides, we evaluated 7-deaza-2´-deoxyguanosine (da^7^G) and 8-aza-7-deaza-2´-deoxyguanosine (a^8^da^7^G) to suppress G-to-A and G-to-T substitutions. Previously reported possible mechanism of G-to-A substitution in the capping step is shown in Fig. 7a. To understand the structural and energetical difference among intermediates, we performed DFT calculations. Geometry optimizations were carried out with the M06-2X functional and with the 6–31 + G(d,p) basis set. The vibrational frequencies were calculated to confirm none of the structures present imaginary frequencies (Fig. S4, Tables S3–S5). The optimized structure of *N*-methyl imidazole adduct of guanine base (G-MeIm), 7-deazaguanine base (da^7^G-MeIm), and 8-aza-7-deazaguanine base (a^8^da^7^G-MeIm) was shown in Fig. 7b. The optimized structure of G-MeIm suggested the formation of C-H···N hydrogen bonding between a hydrogen atom at the C-2 position of methyl imidazole and a nitrogen atom at the N-7 position of guanine base. We hypothesized that this interaction could be eliminated by using 7-deazaguanine base. The dihedral angle of the guanine ring and methyl imidazole ring in G-MeIm was 0.0°. In contrast, that in da^7^G-MeIm and a^8^da^7^G-MeIm were –25.7° and –22.2°, respectively, suggesting disruption of C-H···N hydrogen bonding by eliminating a nitrogen atom at the N-7 position. The calculated energy difference, ΔΔE, of an N-methyl imidazole adduct formation at 7-deazaguanine base and that at guanine base was + 6.6 kcal/mol. In the case of 8-aza-7-deazaguanine base, this value increased to + 13.4 kcal/mol. G-to-T substitution due to 8-oxo-2´-deoxyguanosine formation was expected to get suppressed by using da^7^G or a^8^da^7^G since 8-oxoguanine base would form base pairing with adenine base via Hoogsteen base-pairing.

In our reference sequence, there are three repeat sequences consisting of different dimer steps. To eliminate the effect of systemic errors resulting during chemical synthesis, we used 5´-side repeat sequences as controls and 3´-side sequences as tests (Fig. 7c). The da^7^G and a^8^da^7^G were introduced to three different dimer steps, namely GA, GC, and GT. For synthetic condition, we used the condition as shown in entry 5 in Fig. 6 (Pac_2_O), which showed the highest error rate of G-to-A substitution. It should be noted that 0.02 M iodine was used to compare the effect of non-canonical nucleosides although it is recommended to use CSO for the synthesis of oligonucleotides containing multiple da^7^G.

In the case of 2´-deoxyguanosine (G), the median error rate of G-to-A substitution in control and test was 0.95% and 1.71%, respectively (Fig. 7d). In the case of da^7^G, the median error rate of G-to-A substitution in control and test was 1.90% and 0.18%, respectively. In the case of a^8^da^7^G, the median error rate of G-to-A substitution in control and test was 1.57% and 0.03%, respectively. As we expected, the error rate for G-to-A substitution showed roughly tenfold (Mann–Whitney U test between control and test, *P* = 0.002) and 50-fold decrease (*P* = 0.002) when using da^7^G and a^8^da^7^G, respectively. Although there was a tendency of decrease in G-to-T substitution upon replacement with da^7^G and a^8^da^7^G, we did not observe statistically significant change. Further evaluation will be necessary to discuss the effect of da^7^G and a^8^da^7^G on G-to-T substitution. Interestingly, the error rate of insertion was decreased in a^8^da^7^G (from 0.0011 to 0.0005%, *P* = 0.005), suggesting that the deprotection of DMTr group during the coupling step was suppressed. The protecting group for nucleobases G and da^7^G was isobutyryl, whereas that for a^8^da^7^G was dimethylformamidine (dmf). The dialkyl formamidine protected deoxyadenosines have been reported to be more resistant to depurination than N6-benzoyldeoxyadenosine under acidic conditions. It might be possible that the deprotection of DMTr group during coupling step was inhibited by dmf group. The error rate of deletion in a^8^da^7^G was slightly increased from 0.06 to 0.11% (*P* = 0.009). The reason underlying the generation of deletion product could be insufficient coupling reaction and/or insufficient capping reaction. Since the error rate difference was observed in the same oligonucleotide, the efficiency of capping reaction was expected to be same. Thus, it might be possible that the dmf protected a^8^da^7^G phosphoramidite was slightly unreactive compared to isobutyryl protected deoxyguanosine, suggesting a longer coupling time might be beneficial. We also synthesized oligonucleotide using a^8^da^7^G under a standard synthetic condition (Ac_2_O capping condition). The median error rate of G-to-A substitution in control and test was 0.167% and 0.039%, respectively. In addition, the median error rate of G-to-T substitution was improved from 0.034 to 0.0018%. These data indicated that a^8^da^7^G can improve substitutions even under the Ac_2_O capping condition. From these data, we concluded that a^8^da^7^G can improve the quality of template DNA for genome synthesis.

In summary, we quantified substitutions, insertions, and deletions derived from the chemical synthesis of oligonucleotide (synthetic errors) using next-generation sequencing. Among substitutions, G-to-A substitution was the most prominent followed by G-to-T, C-to-T, T-to-C, and A-to-G substitutions. The observed error rate of G-to-A substitution was influenced by capping conditions, suggesting that the capping step played a major role in the generation of G-to-A substitution. To suppress the synthetic errors, we introduced 7-deaza-2´-deoxyguanosine (da^7^G) and 8-aza-7-deaza-2´-deoxyguanosine (a^8^da^7^G) as error-proof nucleosides. These nucleosides effectively suppressed generation of G-to-A substitution, suggesting that non-canonical nucleosides have the potential to improve the quality of template DNA.

Our method has limitations. The observed error rate is not the actual synthetic errors rate but multiplying them with the read-through efficiency by DNA polymerase. Although this limitation is not important in practical use for genome synthesis, the evaluation of read-through efficiency for each synthetic error will be beneficial to estimate the actual synthetic errors rate. Although the use of non-canonical nucleosides for template DNA synthesis is an effective approach to improve the quality of synthesized sequence, the applications using synthesized oligonucleotide itself, such as nucleic acid therapeutics, will not be applicable. For such purposes, improvement of reaction conditions and protective groups for nucleosides will be also necessary.

Overall, next-generation sequencing of oligonucleotides prepared by assembling reaction is an effective approach for evaluation of synthetic errors derived from chemical synthesis. We believe that our results would contribute to the development of technologies for the synthesis of long DNAs.

## Methods

### General

The natural DNA phosphoramidites and 8-aza-7-deaza-2´-deoxyguanosine phosphoramidite were purchased from Glen Research. 7-Deaza-2´-deoxyguanosine phosphoramidite was purchased from Chem Genes. The synthetic reagents [activators: 1*H*-tetrazole, dicyanoimidazole, 5-benzylthio-1*H*-tetrazole; capping reagents: acetic anhydride, phenoxyacetic anhydride, 1-methylimidiazole; oxidation reagents: iodine, (1S)-(+)-(10-camphorsulfonyl)-oxaziridine; deblocking reagents: dichloroacetic acid; solid support: Glen UnySupport] were all from Glen Research. Anhydrous acetonitrile, molecular sieves 3A, triethylamine, and 3% trichloroacetic acid in dichloromethane were from Fujifilm-Wako. Ammonium hydroxide was from Sigma-Aldrich. Q5 High-Fidelity DNA polymerase and Phusion High-Fidelity DNA polymerase were from New England Biolabs. TaKaRa Ex Taq was from TaKaRa Bio. Indexed adapter oligonucleotides were from Integrated DNA Technologies (IDT). PCR primers were from Eurofins.

### General procedure for oligonucleotide synthesis

Oligonucleotides were synthesized at 1 µmol scale on solid supports of controlled pore glass (CPG) with DNA synthesizer NTS-M (Nihon Techno Service Co., Ltd.) using standard phosphoramidite method. All phosphoramidites were dissolved in anhydrous acetonitrile to prepare 0.1 M solution. The solutions of phosphoramidites and an activator were kept anhydrous by the addition of molecular sieves 3A. The detailed information for all reagents has been provided in the supplementary data. The synthesis was performed with the default setting of 1 µmol scale DNA synthesis in NTS-M unless otherwise noted. The reaction durations for deblocking, coupling, capping, and oxidation steps were 40 s (20 s twice), 20 s (10 s twice), 40 s (20 s twice), and 18 s (10 s and 8 s), respectively. For (1S)-(+)-(10-camphorsulfonyl)-oxaziridine (CSO), the reaction duration for oxidation was 6 min (200 s and 160 s). After oligonucleotide synthesis, DMTr-on CPG solid support was treated with 40% triethylamine in acetonitrile for 30 min to remove the cyanoethyl protecting group from phosphate groups. Cleavage from the CPG solid support was performed using 28% ammonium hydroxide for 1 h at room temperature and removal of the base-protecting group was then carried out for 12–16 h at 55 °C. After the removal of ammonium hydroxide by miVac Duo centrifuge evaporator (Genevac, Ipswich, UK), the crude mixture of oligonucleotides was purified on Sep-Pak Plus C18 cartridge (Waters, US). Oligonucleotides were used without further purification unless otherwise noted.

### Preparation of NGS library and sequencing

To prepare a single strand of complementary sequence of index 1 (i7) adapter, the adapter oligonucleotides (xGen UMI-UDI adapter (IDT), 0.5 µL, 15 µM) was mixed with the primer P2 (5´-CAAGCAGAAGACGGCATACGA, 1.0 µL, 15 µM), 5 µL of pre-mixed 2 × Q5 solution (2 × Q5 buffer, Q5 (0.02U/µL), and dNTP (1 mM)), and water (3.5 µL). After initial heating at 98 °C for 30 s, the reaction was cycled 5 times at: 98 °C for 5 s, 70 °C for 1 s, ramping down (25%) to 50 °C, 50 °C for 30 s, and 72 °C for 20 s. The final extension step was carried out at 72 °C for 3 min. The pre-assembled adapter solution (2.5 µL) was mixed with the solution of synthesized oligonucleotide (1.0 µL, 1 µM), primer P1 (5´-AATGATACGGCGACCACCGA, 15 µM, 2 µL), primer P2 (15 µM, 2 µL), pre-mixed 2 × Q5 solution (50 µL), and water (42.5 µL). After Initial heating at 98 °C for 30 s, the reaction was then cycled 15 times at: 98 °C for 5 s, 70 °C for 1 s, ramping down (25%) to 50 °C, 50 °C for 30 s, and 72 °C for 20 s. The final extension step was carried out at 72 °C for 3 min. The barcoded product was then cleaned using GeneElute PCR clean-up kit (Sigma-Aldrich). The concentrations of barcoded products were determined by qPCR using KAPA Library Quantification kit, according to the provided protocol by the manufacturer. Then, the products were mixed to a final concertation of 1 nM; mixed products were then loaded onto an Illumina iSeq100 (iSeq 100 i1 Reagent v2).

### Pre-processing, alignment, and error parsing

Illumina adapters were trimmed from reads using BBDuk (BBtools: version 38.87). The contaminated reads derived from the PhiX (NC 001,422) or Escherichia coli (U00096.3) genomes were also removed by BBDuk^22^. The paired-end reads were merged as only read pairs having perfectly overlapping regions by BBMerge (BBtools: version 38.87) with pfilter = 1 option. Any merged reads containing N bases and quality scores less than 40 were omitted. Averaged number of reads analyzed in this study was 59,5057.

The alignment and error parsing were performed using in-house Python script, which is slightly modified from Lubock's script^22^. The alignment was performed using a Needleman–Wunsch exhaustive global alignment implemented in the uta-align (version 0.2.0) package from the Python Package Index (PyPI). The settings for match_score, mismatch_score, gap_open_score, and gap_extend_score were 10, − 9, − 15, and − 6, respectively. From the result of alignment, the errors were classified into five classes: M-Mismatch, D-single-base Deletion, I-single-base Insertion, P-multiPle-base deletion, and S-multiple-base inSertion. Each type of error at each position was counted and divided by the total number of reads, and then was multiplied by 100 to calculate the error rates [%]. The relative error frequency (f) per kb was calculated as follows:

$$f = \frac{{\mathop \sum \nolimits_{i} x_{i} \frac{1000}{{l_{i} }}}}{n}$$

where x i is the number of errors in read i, l i is the length of read i, and n is the total number of reads.

## Supplementary Information

Supplementary Information.

## Data Availability

Sequencing data are available from the DDBJ Sequence Read Archive (DRA) with the accession number DRA013805.
