# Supplementary material for: Quantification of synthetic errors during chemical synthesis of DNA and its suppression by non-canonical nucleosides
Source: Sci Rep. 2022 Jul 15;12:12095. doi: 10.1038/s41598-022-16222-2 (PMC9287346; doi:10.1038/s41598-022-16222-2)
Supplement: Supplementary file 1 — Supplementary Information. [file 41598_2022_16222_MOESM1_ESM.pdf]

SUPPLEMENTAL INFORMATION

Quantification of synthetic errors during chemical synthesis of DNA and its suppression by non-canonical nucleosides

Yoshiaki Masaki,[1,2]* Yukiko Onishi,[1] and Kohji Seio [1]

[1]Department of Life Science and Technology, Tokyo Institute of Technology, 4259-J2-16 Nagatsuta, Midori, Yokohama, Kanagawa, 226-8501, JAPAN.

[2]JST, PRESTO, 4-1-8 Honcho, Kawaguchi, Saitama, 332-0012, JAPAN.

*Correspondence: ymasaki@bio.titech.ac.jp

Table of contents

Supplemental experimental information: Catalog ID of reagents............ 3

Figure S1. Reproductivity of synthetic errors under different synthetic conditions............ 5

Figure S2. Boxplots of synthetic errors under different synthetic conditions............ 8

Table S1. Observed mean error frequencies under each synthetic condition............ 11

Table S2. Summary of median error rates under each synthetic condition............ 11

Figure S3. Synthetic condition dependency of error rates............ 12

Figure S4. Optimized structures of guanine base derivatives............ 13

Table S3. Calculated energy of intermediates at M06-2X/6-31+G(d,p)............ 14

Table S4. Frequencies for optimized structures............ 14

Table S5. Cartesian coordinations of calculated nucleobases............ 16

Supplemental experimental information: Catalog ID of reagents

Phosphoramidites

- dT-CE phosphoramidite (Glen research)
  - 10-1030-2C — 5'-Dimethoxytrityl-2'-deoxyThymidine,3'-[(2-cyanoethyl)-(N,N-diisopropyl)]-phosphoramidite
- dG-CE phosphoramidite (Glen research)
  - 10-1020-2C — 5'-Dimethoxytrityl-N-isobutyryl-2'-deoxyGuanosine,3'-[(2-cyanoethyl)-(N,N-diisopropyl)]-phosphoramidite
- Ac-dC-CE phosphoramidite (Glen research)
  - 10-1015-2C — 5'-Dimethoxytrityl-N-acetyl-2'-deoxyCytidine,3'-[(2-cyanoethyl)-(N,N-diisopropyl)]-phosphoramidite
- dA-CE phosphoramidite (Glen research)
  - 10-1000-2C — 5'-Dimethoxytrityl-N-benzoyl-2'-deoxyAdenosine,3'-[(2-cyanoethyl)-(N,N-diisopropyl)]-phosphoramidite
- 7-Deaza deoxy Guanosine CED phosphoramidite (Chem Genes)
  - ANP-4857 — 5'-Dimethoxytrityl-N-isobutyryl-7-deaza-2'-deoxyGuanosine,3'-[(2-cyanoethyl)-(N,N-diisopropyl)]-phosphoramidite
- 7-Deaza-8-aza-dG-CE Phosphoramidite (Glen research)
  - 10-1073-90E — 5'-Dimethoxytrityl-N-dimethylformamidine-8-aza-7-deaza-2'-deoxyGuanosine,3'-[(2-cyanoethyl)-(N,N-diisopropyl)]-phosphoramidite

Activators

- 1*H*-tetrazole (Glen research)
  - 30-3102-52 — Sublimed 1*H*-Tetrazole in Anhydrous Acetonitrile
- DCI (Glen research)
  - 30-3152-52 — 0.25M DCI in Anhydrous Acetonitrile
- BTT (Glen research)
  - 30-3172-52 — 0.25M 5-Benzylthio-1*H*-Tetrazole (BTT) in Acetonitrile

Capping reagents

- Ac_2O, Py, MeIm/THF (Glen research)
  - 40-4012-52 — 10% Acetic Anhydride in Tetrahydrofuran
  - 40-4122-52 — 10% 1-Methylimidazole, 10 % Pyridine in Tetrahydrofuran
- Ac_2O, 2,6-lutidine, MeIm/THF (Glen research)
  - 40-4010-52 — 10% Acetic Anhydride, 10% 2,6-Lutidine in Tetrahydrofuran
  - 40-4220-52 — 16% 1-Methylimidazole in Tetrahydrofuran
- Pac_2O, Py, MeIm/THF (Glen research)
  - 40-4212-52 — 5% Phenoxyacetic anhydride/Tetrahydrofuran (5:95, v/v/v)
  - 40-4122-52 — 10% 1-Methylimidazole in Tetrahydrofuran/Pyridine

Oxidation reagents

- 0.02 M I_2 (Glen research)
  - 40-4132-52 — 0.02M Iodine in Tetrahydrofuran/ Water/ Pyridine

0.5 M CSO (Glen research)

40-4632-52E 0.5M CSO in Anhydrous Acetonitrile

Deblocking reagents

3% TCA (Fujifilm-Wako)

042-28921 3w/v% Trichloroacetic Acid, Dichloromethane Solution

DCA (Glen research)

40-4040-57 3% Dichloroacetic acid in Dichloromethane

Solid support

Glen UnySupport™ 1000

20-5241-41 Glen UnySupport™ 1000

1*H*-tetrazole, 10%Ac_2O -10%MeIm/10%Py/THF, 0.02M I_2, 3%TCA

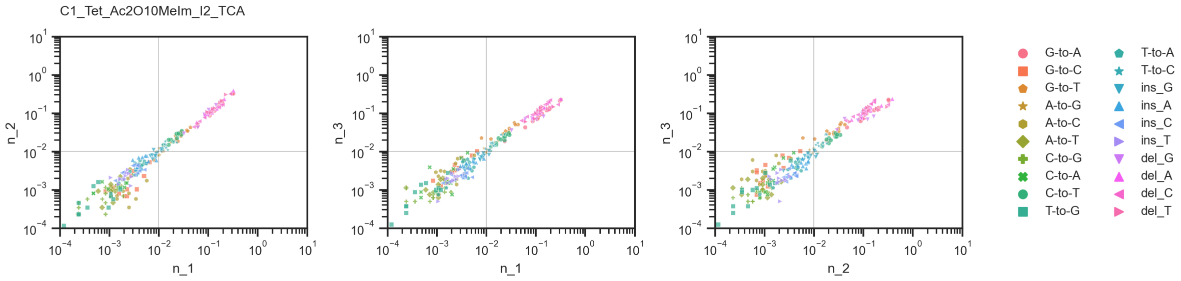

DCI, 10%Ac_2O -10%MeIm/10%Py/THF, 0.02M I_2, 3%TCA

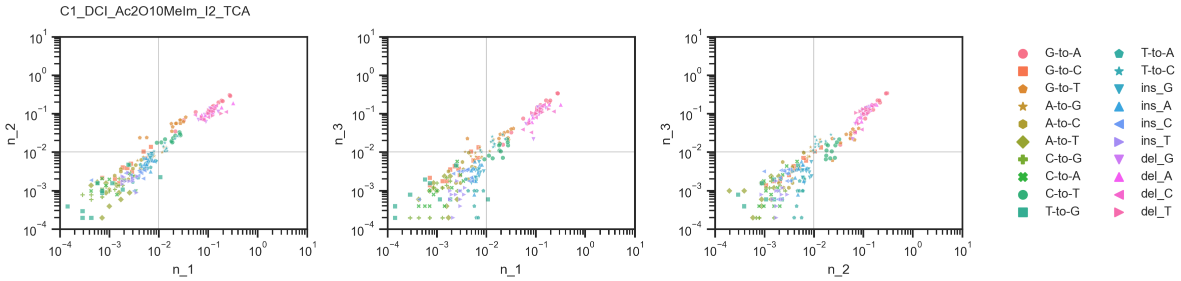

BTT, 10%Ac_2O -10%MeIm/10%Py/THF, 0.02M I_2, 3%TCA

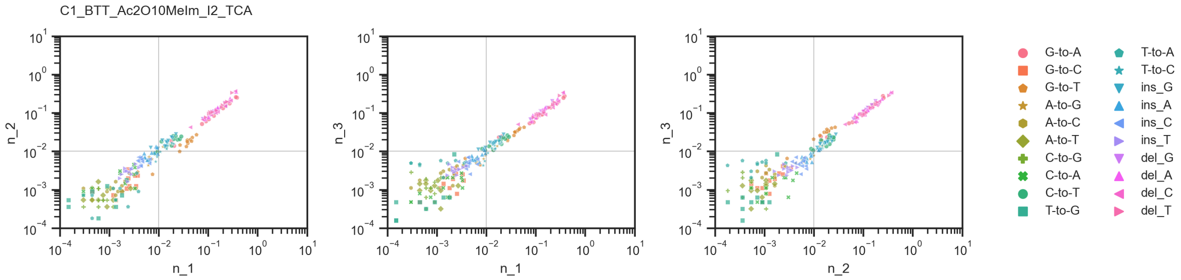

BTT, 10%Ac_2O 10%Lut -16%MeIm /THF, 0.02M I_2, 3%TCA

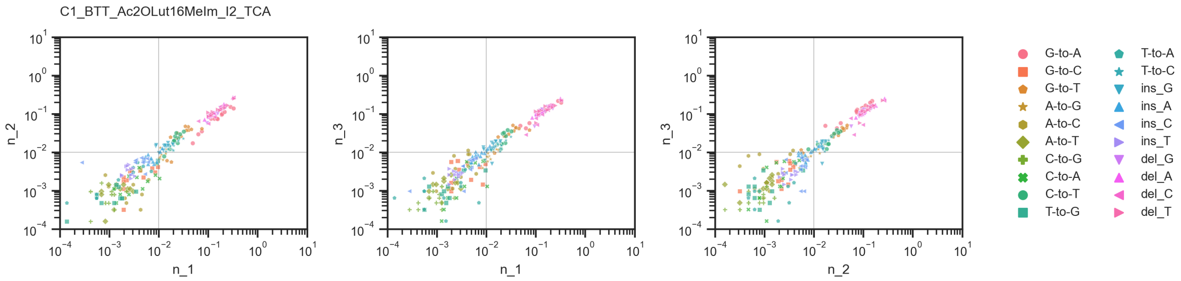

Figure S1. Reproductivity of synthetic errors under different synthetic conditions.

BTT, 5%Pac_2O -10%MeIm/10%Py/THF, 0.02M I_2, 3%TCA

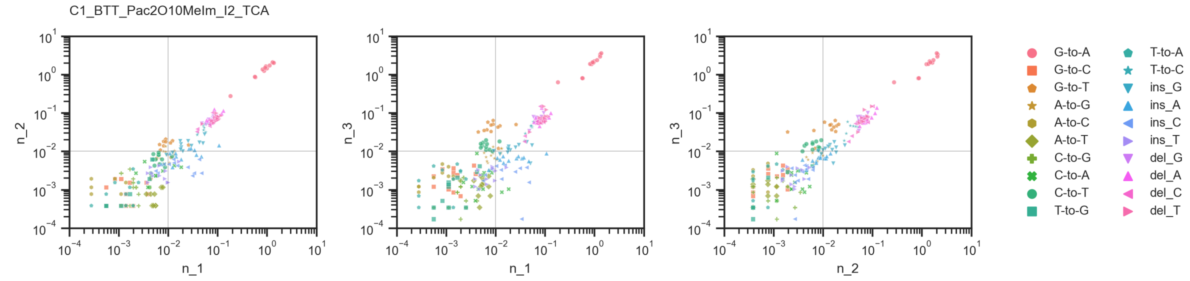

1*H*-tetrazole, 10%Ac_2O -10%MeIm/10%Py/THF, CSO, 3%TCA

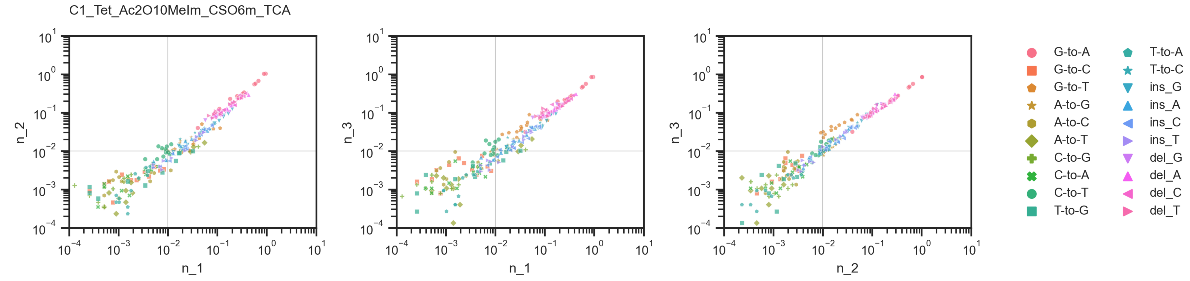

1*H*-tetrazole, 10%Ac_2O -10%MeIm/10%Py/THF, 0.02M I_2, 3%DCA

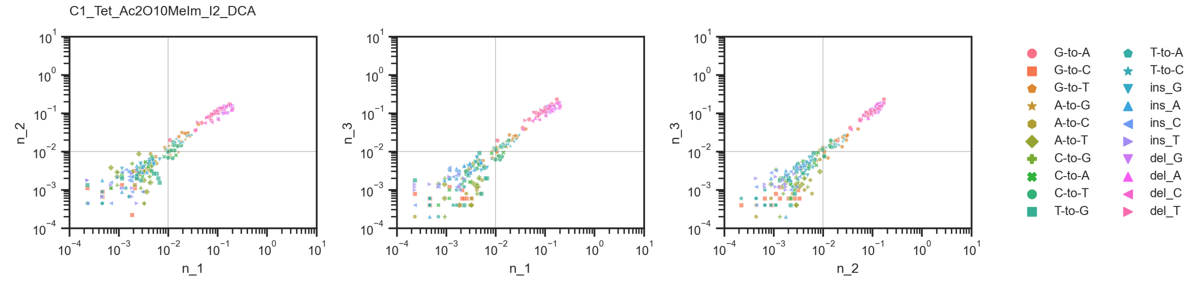

1*H*-tetrazole, 10%Ac_2O -10%MeIm/10%Py/THF, 0.02M I_2, 3%TCAx2

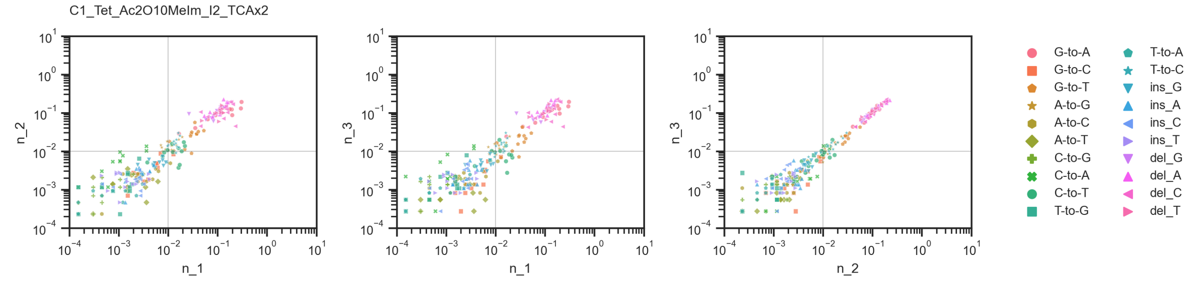

Figure S1. Continued.

1*H*-tetrazole, 10%Ac_2O -10%MeIm/10%Py/THF, 0.02M I_2, 3%TCA, **Phusion**

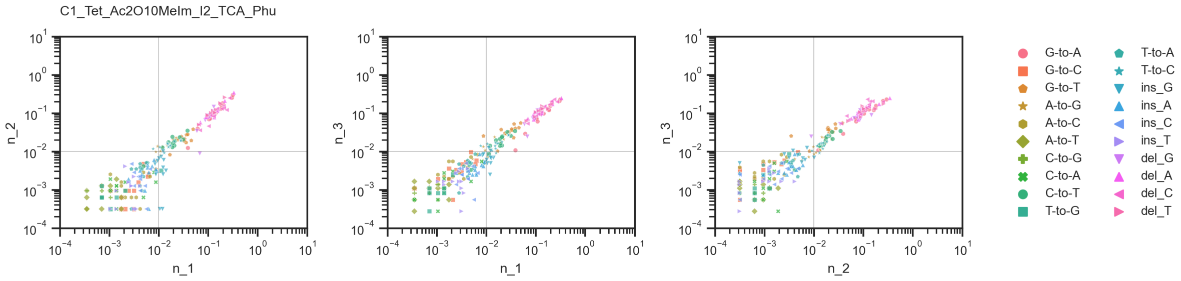

1*H*-tetrazole, 10%Ac_2O -10%MeIm/10%Py/THF, 0.02M I_2, 3%TCA, **Takara Ex Taq**

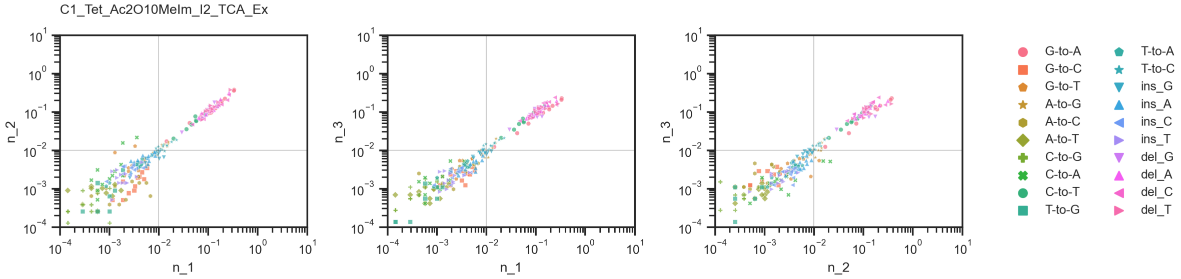

BTT, 5%Pac_2O -10%MeIm/10%Py/THF, 0.02M I_2, 3%TCA,**da^{7}dG**

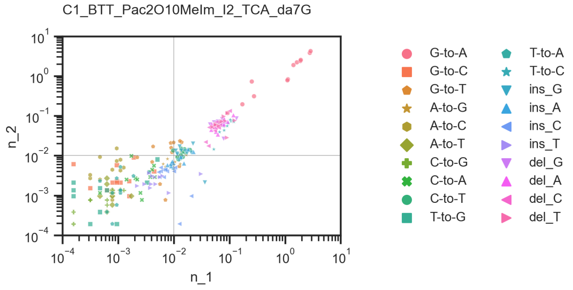

BTT, 5%Pac_2O -10%MeIm/10%Py/THF, 0.02M I_2, 3%TCA,**a^{8}da^{7}dG**

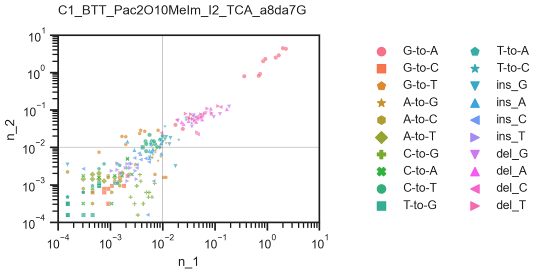

Figure S1. Continued.

1*H*-tetrazole, 10%Ac_2O -10%MeIm/10%Py/THF, 0.02M I_2, 3%TCA

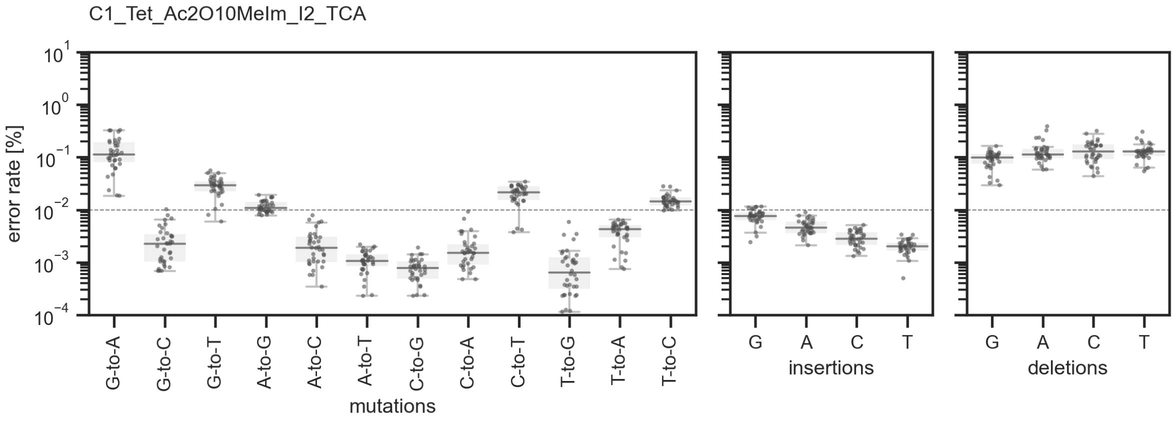

DCI, 10%Ac_2O -10%MeIm/10%Py/THF, 0.02M I_2, 3%TCA

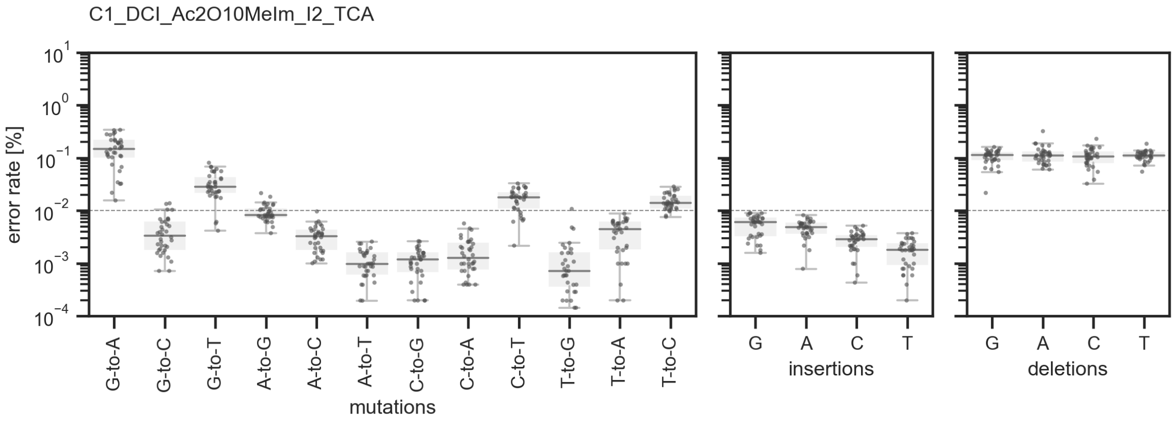

BTT, 10%Ac_2O -10%MeIm/10%Py/THF, 0.02M I_2, 3%TCA

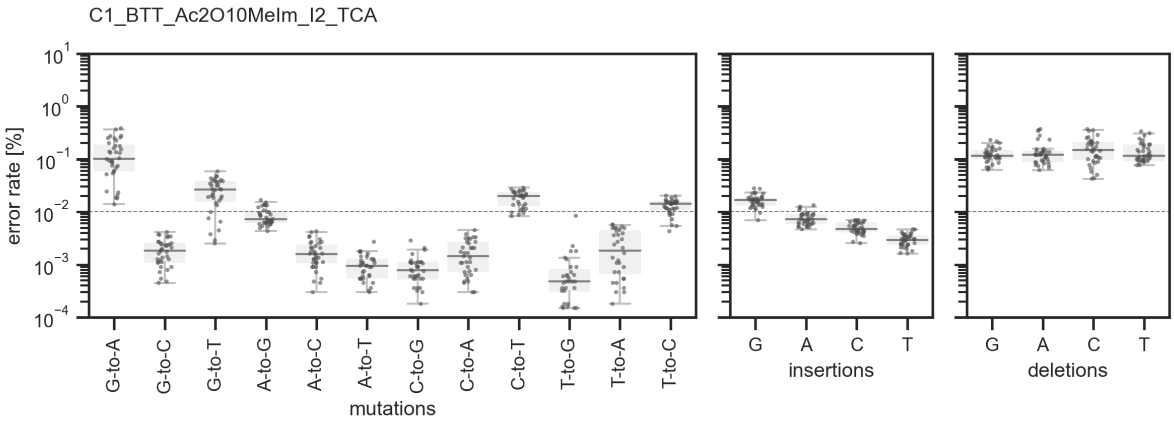

Figure S2. Boxplots of synthetic errors under different synthetic conditions

BTT, 10%Ac_2O 10%Lut -16%MeIm /THF, 0.02M I_2, 3%TCA

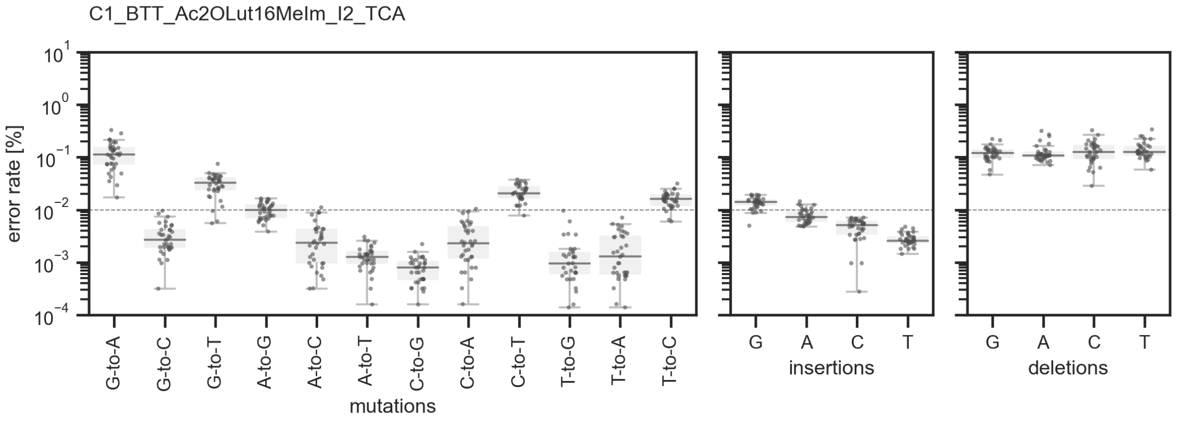

BTT, 5%Pac_2O -10%MeIm/10%Py/THF, 0.02M I_2, 3%TCA

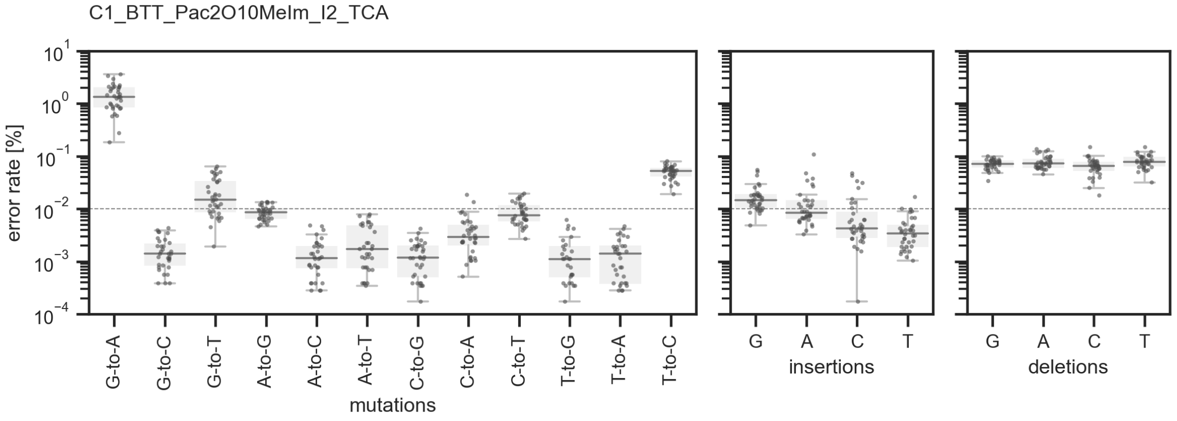

1*H*-tetrazole, 10%Ac_2O -10%MeIm/10%Py/THF, CSO, 3%TCA

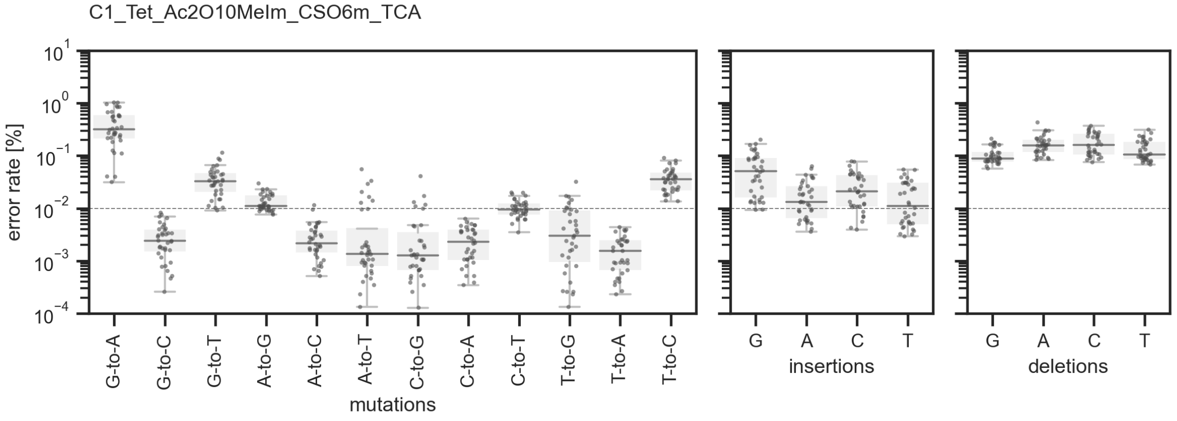

Figure S2. Continued.

1*H*-tetrazole, 10%Ac_2O -10%MeIm/10%Py/THF, 0.02M I_2, 3%DCA

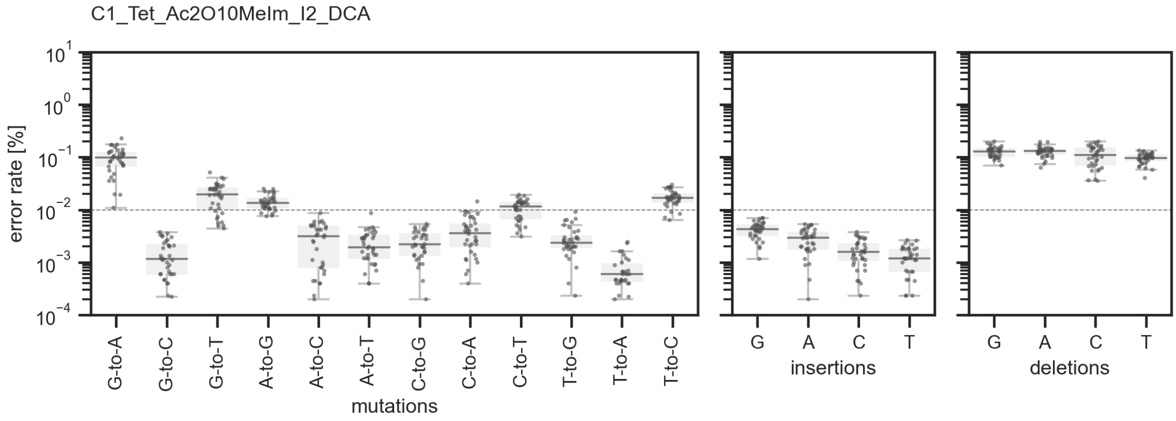

1*H*-tetrazole, 10%Ac_2O -10%MeIm/10%Py/THF, 0.02M I_2, 3%TCAx2

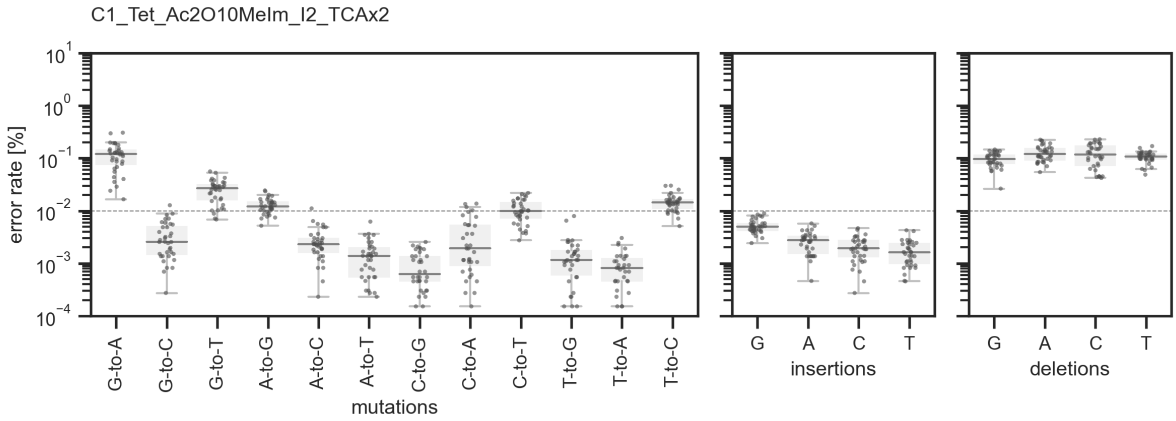

Figure S2. Continued.

Table S1. Observed mean error frequencies under each synthetic condition.

The unit of data in the table is nt per kb. Standard deviations were derived from three independent experiment.

activator	1*H*-Tetrazole	1*H*-Tetrazole	1*H*-Tetrazole	DCI	BTT	BTT	BTT	1*H*-Tetrazole	1*H*-Tetrazole	1*H*-Tetrazole
cap A	Ac_2O	Ac_2O	Ac_2O	Ac_2O	Ac_2O	Ac_2O/Lut	Pac_2O	Ac_2O	Ac_2O	Ac_2O
cap B	10%MeIm/Py	10%MeIm/Py	10%MeIm/Py	10%MeIm/Py	10%MeIm/Py	16%MeIm	10%MeIm/Py	10%MeIm/Py	10%MeIm/Py	10%MeIm/Py
oxidation	0.02 M I_2	0.02 M I_2	0.02 M I_2	0.02 M I_2	0.02 M I_2	0.02 M I_2	0.02 M I_2	CSO	0.02 M I_2	0.02 M I_2
deblocking	3%TCA	3%TCA	3%TCA	3%TCA	3%TCA	3%TCA	3%TCA	3%TCA	3%DCA	3%TCA x 2
polymerase	Ex	Phu	Q5	Q5	Q5	Q5	Q5	Q5	Q5	Q5
error frequency	2.067 ± 0.168	1.993 ± 0.299	2.064 ± 0.129	2.023 ± 0.134	2.334 ± 0.305	2.299 ± 0.347	4.951 ± 1.278	3.303 ± 0.386	1.793 ± 0.055	1.881 ± 0.054
single substitution	0.641 ± 0.077	0.550 ± 0.075	0.566 ± 0.039	0.620 ± 0.076	0.519 ± 0.101	0.539 ± 0.126	3.908 ± 1.436	1.358 ± 0.108	0.441 ± 0.020	0.494 ± 0.071
single insertion	0.044 ± 0.003	0.042 ± 0.017	0.044 ± 0.004	0.038 ± 0.011	0.080 ± 0.008	0.073 ± 0.003	0.116 ± 0.085	0.320 ± 0.095	0.025 ± 0.004	0.030 ± 0.002
single deletion	1.236 ± 0.078	1.233 ± 0.139	1.273 ± 0.073	1.138 ± 0.049	1.464 ± 0.183	1.340 ± 0.194	0.756 ± 0.078	1.523 ± 0.192	1.194 ± 0.045	1.166 ± 0.026
multiple insertion	0.0003 ± 0.0001	0.0004 ± 0.00002	0.0005 ± 0.0001	0.0007 ± 0.0002	0.0008 ± 0.0001	0.0011 ± 0.0007	0.0006 ± 0.0003	0.0015 ± 0.0003	0.0005 ± 0.0002	0.0006 ± 0.0002
multiple deletion	0.145 ± 0.011	0.168 ± 0.069	0.180 ± 0.014	0.226 ± 0.081	0.271 ± 0.034	0.345 ± 0.038	0.171 ± 0.037	0.100 ± 0.001	0.132 ± 0.001	0.190 ± 0.008

Table S2. Summary of median error rates under each synthetic condition.

The unit of data in the table is %. Sum of error rates including substitutions, insertions, and deletions were divided by 4 to estimate the error rate per nt.

activator	1*H*-Tetrazole	1*H*-Tetrazole	1*H*-Tetrazole	DCI	BTT	BTT	BTT	1*H*-Tetrazole	1*H*-Tetrazole	1*H*-Tetrazole
cap A	Ac_2O	Ac_2O	Ac_2O	Ac_2O	Ac_2O	Ac_2O/Lut	Pac_2O	Ac_2O	Ac_2O	Ac_2O
cap B	10%MeIm/Py	10%MeIm/Py	10%MeIm/Py	10%MeIm/Py	10%MeIm/Py	16%MeIm	10%MeIm/Py	10%MeIm/Py	10%MeIm/Py	10%MeIm/Py
oxidation	0.02 M I_2	0.02 M I_2	0.02 M I_2	0.02 M I_2	0.02 M I_2	0.02 M I_2	0.02 M I_2	CSO	0.02 M I_2	0.02 M I_2
deblocking	3%TCA	3%TCA	3%TCA	3%TCA	3%TCA	3%TCA	3%TCA	3%TCA	3%DCA	3%TCA x 2
polymerase	Ex	Phu	Q5	Q5	Q5	Q5	Q5	Q5	Q5	Q5
G-to-A	0.116	0.107	0.113	0.149	0.101	0.113	1.333	0.318	0.099	0.120
G-to-C	0.002	0.002	0.002	0.003	0.002	0.003	0.001	0.002	0.001	0.003
G-to-T	0.004	0.028	0.029	0.028	0.026	0.033	0.015	0.033	0.020	0.027
A-to-G	0.011	0.011	0.011	0.008	0.007	0.010	0.009	0.011	0.014	0.012
A-to-C	0.001	0.002	0.002	0.003	0.002	0.002	0.001	0.002	0.003	0.002
A-to-T	0.001	0.001	0.001	0.001	0.001	0.001	0.001	0.001	0.002	0.001
C-to-G	0.000	0.001	0.001	0.001	0.001	0.001	0.001	0.001	0.002	0.001
C-to-A	0.002	0.001	0.002	0.001	0.001	0.002	0.003	0.002	0.004	0.002
C-to-T	0.076	0.019	0.021	0.018	0.019	0.020	0.007	0.009	0.011	0.010
T-to-G	0.000	0.001	0.001	0.001	0.000	0.001	0.001	0.003	0.002	0.001
T-to-A	0.004	0.004	0.004	0.004	0.001	0.001	0.001	0.001	0.000	0.001
T-to-C	0.013	0.015	0.014	0.014	0.014	0.016	0.052	0.036	0.017	0.014
ins_G	0.008	0.007	0.008	0.006	0.016	0.014	0.014	0.050	0.004	0.005
ins_A	0.005	0.004	0.005	0.005	0.007	0.007	0.008	0.013	0.003	0.003
ins_C	0.003	0.003	0.003	0.003	0.005	0.005	0.004	0.021	0.002	0.002
ins_T	0.002	0.002	0.002	0.002	0.003	0.003	0.003	0.011	0.001	0.002
del_G	0.106	0.091	0.099	0.113	0.116	0.120	0.071	0.089	0.129	0.097
del_A	0.109	0.112	0.113	0.110	0.120	0.108	0.073	0.158	0.130	0.121
del_C	0.129	0.125	0.128	0.107	0.146	0.126	0.065	0.159	0.109	0.117
del_T	0.118	0.124	0.127	0.111	0.116	0.125	0.078	0.106	0.097	0.108
error rate per nt	0.177	0.165	0.171	0.172	0.176	0.178	0.436	0.257	0.163	0.162
purity per nt	99.82	99.83	99.83	99.83	99.82	99.82	99.56	99.74	99.84	99.84

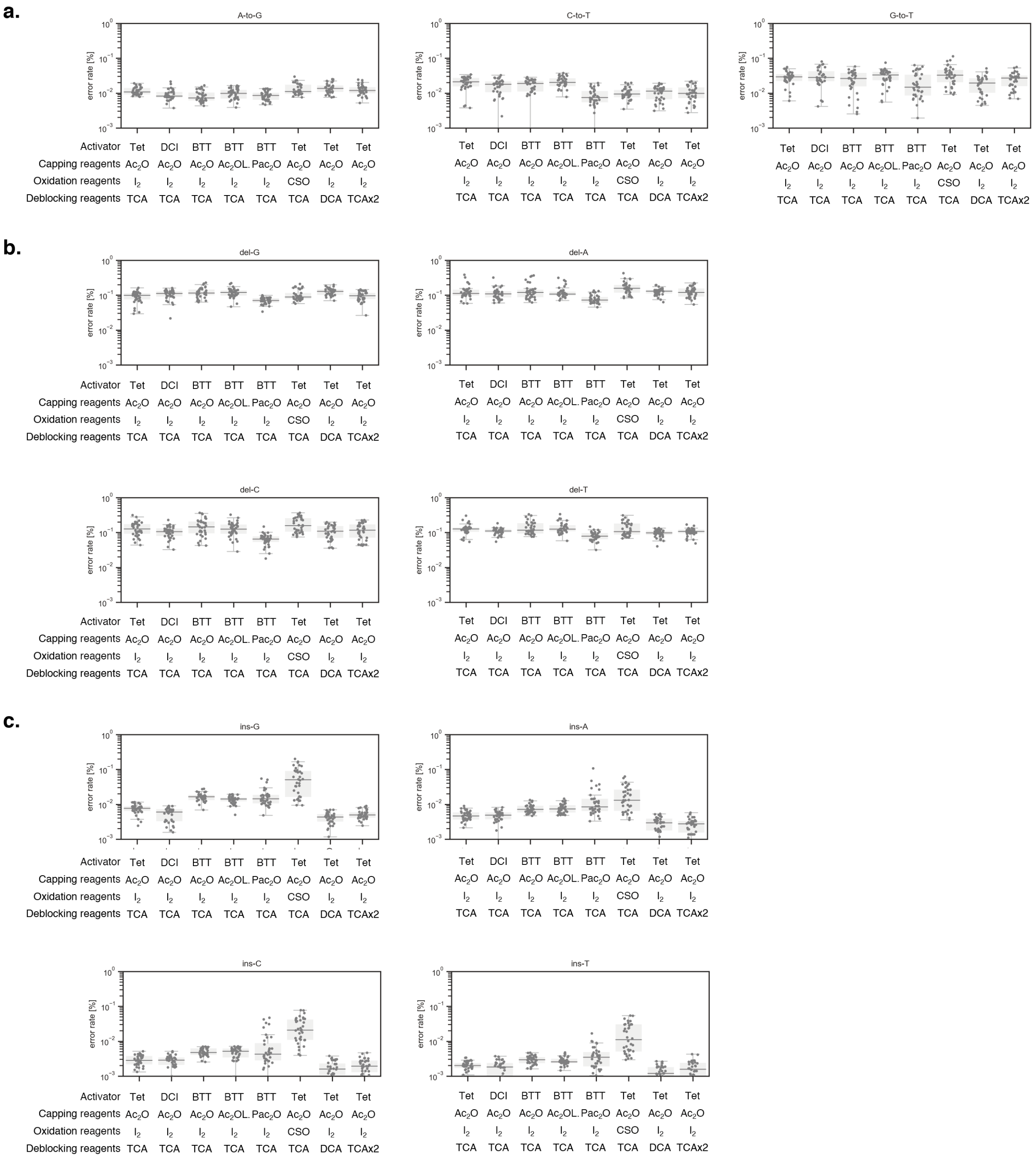

Figure S3. Synthetic condition dependency of error rates.

(**a**) The other substitutions having median error rates more than 0.01%. (**b**) Deletions. (**c**) Insertions.

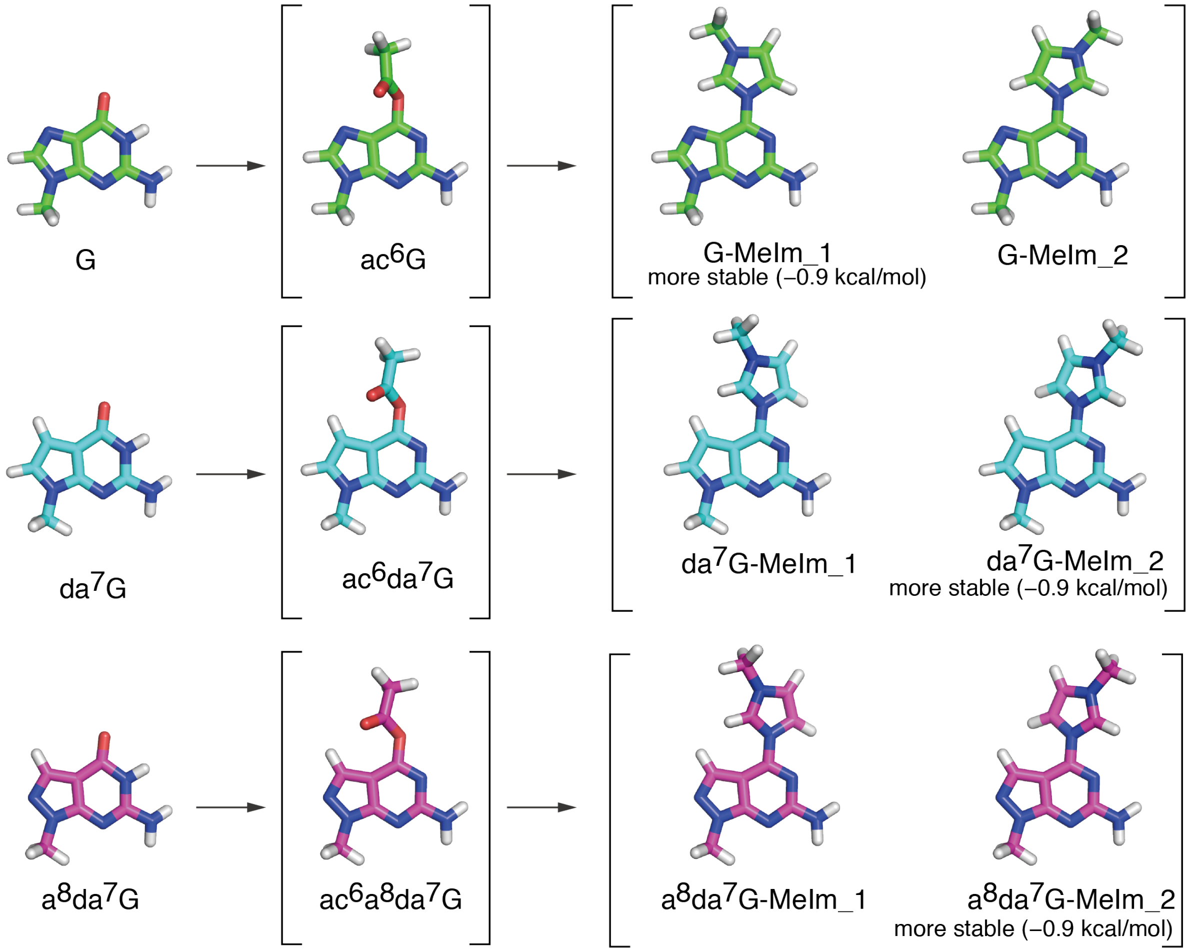

$\Delta\Delta E$_da^{7}G = $\Delta E_{(\text{da7G-MeIm\_2 – da7G})} - \Delta E_{(\text{G-MeIm 1 – G})}$ = +6.6 kcal/mol

$\Delta\Delta E$_a^{8}da^{7}G = $\Delta E_{(\text{a8da7G-MeIm\_2 – a8da7G})} - \Delta E_{(\text{G-MeIm 1 – G})}$ = +13.4 kcal/mol

Figure S4. Optimized structures of guanine base derivatives.

Geometry optimization were carried out with the M06-2X functional and with the 6-31+G(d,p) basis set. The vibrational frequencies were calculated to confirm none of the structures presents imaginary frequencies. Calculations were done with Gaussian 16 RevA.03.

Table S3. Calculated energy of intermediates at M06-2X/6-31+G(d,p).

	E [au]	dE [au]	dE_conf [kcal/mol]	ddE [kcal/mol]	
G	-581.67239242	-			
G_ac	-734.26266357	-152.59027115			
G_MeIm_1	-771.09937107	-189.42697865	-0.9		
G_MeIm_2	-771.09800187	-189.42560945			
da7G	-565.63145866	-			
da7G_ac	-718.21879528	-152.58733662		1.8	from G_ac
da7G_MeIm_1	-755.04644286	-189.41498420		7.5	from G_MeIm_1
da7G_MeIm_2	-755.04792713	-189.41646847	-0.9	6.6	from G_MeIm_1
a8da7G	-581.65833716	-			
a8da7G_ac	-734.24321477	-152.58487761		3.4	from G_ac
a8da7G_MeIm_1	-771.06249366	-189.40415650		14.3	from G_MeIm_1
a8da7G_MeIm_2	-771.06388291	-189.40554575	-0.9	13.4	from G_MeIm_1

Table S4. Frequencies for optimized structures.

Frequencies for G				Frequencies for G-MeIm_1				Frequencies for G-MeIm_2		
49.9758	107.3518	144.6734		51.3048	62.345	73.9702		41.1419	60.6907	71.6343
176.4399	210.8781	249.1699		86.9442	110.05	130.894		88.488	113.3929	123.8435
322.604	338.0129	365.8631		154.9718	185.5455	216.6829		145.019	161.0676	213.3834
384.0529	488.3383	523.204		224.6286	271.0705	278.0629		224.6022	267.2009	273.8848
567.9461	602.3205	626.8031		298.6553	307.2878	355.0932		295.1552	316.4345	354.2198
661.5456	684.9338	706.9515		406.6622	424.626	520.4509		412.94	420.7731	513.8539
741.1092	743.1049	772.5257		525.0672	564.0757	617.7199		526.5401	565.0384	616.9981
848.3656	871.1324	1044.806		640.0045	645.4854	682.9235		639.0144	643.8688	675.5531
1073.4866	1092.0922	1120.669		688.6357	702.2994	738.9079		689.081	705.7851	737.3387
1152.1716	1173.7736	1260.8072		748.7123	786.5995	808.2978		749.2946	792.7913	808.6239
1323.0201	1345.1758	1399.2611		866.1672	877.8126	908.9966		864.8836	877.5624	886.4497
1430.1911	1457.854	1488.0281		911.4263	919.8684	1036.2264		912.6205	933.1813	1045.2564
1499.7399	1520.1757	1549.7902		1044.0249	1067.5162	1071.6786		1051.0461	1054.6911	1068.8378
1603.6632	1636.077	1641.0902		1093.8305	1107.9233	1118.2899		1093.944	1105.8277	1140.3672
1696.0271	1858.5978	3091.4263		1154.2546	1155.1609	1156.5996		1147.9171	1153.7371	1154.5717
3169.5579	3190.333	3277.1237		1238.3558	1254.0476	1287.9177		1241.2254	1252.6399	1284.5604
3610.7082	3631.9676	3730.54		1339.2432	1350.409	1388.1746		1336.4099	1353.3679	1375.807
				1404.2385	1427.8602	1449.4083		1400.6582	1426.6224	1451.3719
				1457.5004	1464.1049	1487.5611		1461.4782	1464.2384	1487.8382
				1492.3492	1493.0408	1508.0051		1491.1377	1493.7539	1503.0323
				1519.3057	1522.952	1536.0005		1518.9553	1522.3201	1535.1882
				1596.5092	1611.3525	1653.5075		1597.6306	1616.0653	1646.9395
				1658.6017	1670.628	1747.9184		1663.2271	1669.637	1749.8324
				3100.6614	3109.5805	3187.1705		3100.8726	3108.7648	3186.4185
				3202.6606	3202.7488	3215.8645		3201.1932	3203.5408	3215.7324
				3270.9411	3281.802	3319.626		3281.5839	3299.0356	3326.1279
				3346.8202	3653.5389	3796.2449		3330.4113	3654.5554	3797.7799

Table S4. continued.

Frequencies for da7G				Frequencies for da7G-MeIm_1				Frequencies for da7G-MeIm_2		
34.253	108.9805	145.9707		41.2369	46.2949	60.7219		40.4374	47.052	65.123
177.0172	225.6399	231.2491		80.9923	92.2125	133.4315		80.1747	103.3641	130.9676
312.8236	338.2144	360.2562		160.097	203.4719	223.0813		160.3787	215.8964	232.9064
386.03	487.3668	523.654		236.7927	252.0076	273.3859		246.995	260.7644	267.4154
585.7207	592.1861	619.486		297.7726	309.9122	350.4194		297.8282	320.1194	350.4444
627.2053	675.8237	695.3857		404.8217	423.2039	509.3541		410.7539	420.3413	501.999
725.2834	738.0749	741.6069		519.6482	564.6981	601.4059		520.7826	565.2172	601.0345
787.3654	867.5915	890.8131		624.0344	639.3064	677.2388		623.1506	639.1221	670.8293
1025.8614	1048.2239	1084.6464		690.6835	701.9272	716.8822		692.8463	702.2151	715.3859
1111.8365	1125.9692	1153.2598		724.3173	752.3439	782.5646		726.8942	753.7012	776.8116
1183.4063	1264.788	1304.386		814.4505	857.7343	867.6227		813.968	857.3132	871.1328
1338.5665	1394.6535	1456.0529		871.1256	904.3051	912.6532		884.719	901.5824	909.4452
1468.2666	1504.6831	1510.5624		1023.3931	1043.1015	1048.7646		1025.8161	1047.0303	1053.5582
1521.7271	1559.8276	1599.0727		1068.6817	1102.6791	1107.5985		1058.6779	1105.1813	1105.921
1621.898	1644.5379	1704.9032		1123.3487	1126.2027	1153.3235		1122.8147	1132.1237	1150.8435
1832.7582	3081.3407	3151.3169		1153.5716	1155.009	1225.0504		1153.6528	1155.322	1226.3694
3198.1319	3288.8586	3311.0892		1262.806	1286.6733	1320.776		1264.3793	1280.2959	1320.2071
3605.697	3637.4636	3723.4791		1352.6256	1388.7109	1419.2851		1360.4025	1380.351	1419.0004
				1433.1447	1446.2395	1456.139		1431.5501	1451.5395	1458.2985
				1463.7094	1485.1646	1490.6901		1463.4431	1480.3875	1491.4659
				1503.5513	1509.5605	1518.0023		1503.4155	1508.9249	1517.6388
				1523.0212	1552.199	1605.9202		1522.5281	1551.5428	1605.8279
				1616.1027	1639.6236	1647.9096		1615.8695	1632.3426	1650.8082
				1667.5334	1737.1061	3094.9071		1666.8471	1737.6897	3094.7836
				3108.8905	3174.4794	3202.1229		3109.6584	3174.2394	3202.8499
				3207.7131	3214.7095	3286.8377		3207.4977	3216.2418	3289.2242
				3304.5053	3318.8023	3322.6031		3310.9761	3319.4512	3326.8969
				3340.8179	3651.8845	3793.3825		3344.4409	3650.1436	3789.7876

Frequencies for a8da7G				Frequencies for a8da7G-MeIm_1				Frequencies for a8da7G-MeIm_2		
73.5188	111.0199	145.3622		40.6916	59.4687	68.5314		38.2043	62.2313	74.4392
173.213	225.9911	238.1074		79.7515	90.9313	130.926		80.1323	102.4009	129.406
329.1028	338.7956	362.9981		157.1758	222.1551	234.712		156.5269	216.4173	232.2487
387.8539	488.277	529.1823		236.4183	257.8346	274.4756		237.4785	259.1624	265.981
548.194	591.7227	627.8194		297.6274	307.7544	353.3275		297.1995	319.4849	353.1454
658.8923	677.7849	704.1189		406.0111	424.3093	528.6537		411.7414	421.6779	528.4784
742.3137	749.4625	777.7412		535.4491	560.5393	621.7393		531.1892	560.7738	621.01
886.0476	898.975	1020.2702		638.8761	651.1132	676.7675		639.3199	650.609	671.8324
1055.7424	1093.9421	1124.229		692.8413	701.7228	736.6684		693.8037	700.1354	736.3442
1155.8514	1182.2865	1244.544		743.5914	783.1738	806.5338		743.4409	773.9636	805.688
1295.0712	1331.7404	1354.9077		848.5003	861.6789	865.1027		854.8583	862.5971	885.1776
1451.9765	1474.8116	1492.8494		888.6696	914.5822	1018.6699		889.1364	901.361	1019.1229
1502.4678	1522.5809	1533.1661		1044.3123	1049.3703	1063.279		1046.5676	1047.5838	1062.6816
1607.2235	1628.6466	1650.5261		1103.7334	1107.228	1124.9841		1105.3895	1106.0792	1130.7496
1703.634	1846.6379	3093.2042		1153.788	1154.6456	1154.8495		1151.3208	1154.8872	1155.4762
3172.2083	3201.7805	3296.5445		1220.2846	1272.1926	1291.6536		1220.3384	1275.556	1286.7349
3615.5288	3636.6489	3737.4898		1310.1247	1327.5684	1377.8571		1308.2592	1332.2442	1379.87
				1395.2784	1432.9445	1451.7533		1386.8604	1432.026	1454.8128
				1455.3465	1464.4141	1490.3353		1460.0537	1464.5235	1490.6879
				1491.3229	1495.4749	1503.8941		1490.9866	1491.7368	1504.4434
				1522.1792	1525.9206	1551.1502		1521.6836	1526.0043	1553.4709
				1580.1525	1612.7054	1636.607		1577.9613	1614.0093	1628.9805
				1651.8392	1676.2453	1739.5489		1653.3838	1675.145	1740.0635
				3102.3333	3109.3517	3188.9866		3103.115	3109.8565	3189.6773
				3203.6867	3210.9578	3215.4773		3204.0604	3211.6762	3216.4563
				3282.4911	3317.6414	3321.5165		3289.7015	3319.1723	3324.1988
				3342.6348	3649.783	3792.4202		3344.6057	3650.7605	3793.0203

Table S5. Cartesian coordinations of calculated nucleobases

Cartesian coordinate of G

N	-1.95736	0.23697	-0.00654
N	-2.50639	-2.02861	-0.06510
N	-0.27267	-1.41321	0.01022
N	1.39851	1.77738	0.01044
N	1.92452	-0.40418	-0.00088
C	2.71628	-1.61872	-0.00814
C	-1.52941	-1.06319	-0.00211
C	0.56297	-0.33877	0.00185
C	0.25467	1.01601	0.01011
C	-1.13271	1.40491	0.00192
C	2.36695	0.90244	0.00426
O	-1.65519	2.49944	-0.00731
H	2.50134	-2.21284	0.88219
H	3.77130	-1.34366	-0.01640
H	-2.94650	0.44111	-0.08733
H	-3.38460	-1.83458	0.39421
H	-2.15010	-2.96067	0.09485
H	3.42439	1.13143	0.00271
H	2.48699	-2.21096	-0.89604

Cartesian coordinate of G-MeIm_1

N	-0.31703	1.77815	0.00003
N	-2.11864	3.20865	-0.00024
N	-2.58143	0.95487	0.00008
N	-0.47516	-1.93562	-0.00003
N	-2.68047	-1.46188	0.00002
C	-4.12383	-1.66040	0.00000
C	-1.66865	1.93878	0.00004
C	-2.04276	-0.25273	0.00003
C	-0.66982	-0.56816	-0.00002
C	0.14968	0.55041	-0.00001
C	-1.69187	-2.41783	0.00002
N	1.57377	0.42194	-0.00001
H	-4.55754	-1.20348	0.89052
H	-4.32645	-2.73094	0.00034
H	-1.46839	3.97637	0.00087
H	-3.11092	3.38103	0.00076
H	-1.93885	-3.47176	0.00002
H	-4.55747	-1.20404	-0.89084
C	2.47547	1.46929	-0.00011
C	2.24504	-0.74014	0.00005
C	3.71336	0.91055	-0.00013
N	3.54414	-0.46282	-0.00001
C	4.62655	-1.45083	0.00006
H	2.13700	2.49245	-0.00018
H	1.77685	-1.71775	0.00011
H	4.69297	1.36219	-0.00021
H	5.23439	-1.31644	0.89486
H	5.23449	-1.31645	-0.89468
H	4.18859	-2.44769	0.00004

Table S5. continued.

Cartesian coordinate of G-MeIm_2

N	0.03561	1.55953	-0.00010
N	-1.37463	3.37943	0.00045
N	-2.35747	1.30049	-0.00006
N	-1.00706	-2.01237	0.00003
N	-3.03322	-1.01819	0.00002
C	-4.48171	-0.86379	0.00002
C	-1.23616	2.03844	-0.00003
C	-2.12360	-0.00021	-0.00001
C	-0.86350	-0.63857	0.00001
C	0.19650	0.25221	-0.00006
C	-2.30232	-2.18551	0.00006
N	1.55188	-0.20405	-0.00008
H	-4.79336	-0.31626	0.89056
H	-4.93533	-1.85443	0.00052
H	-0.56376	3.97445	-0.00181
H	-2.29913	3.77896	-0.00146
H	-2.79648	-3.14874	0.00009
H	-4.79349	-0.31711	-0.89100
C	1.98210	-1.52034	-0.00028
C	2.61710	0.60775	0.00017
C	3.34039	-1.47791	-0.00015
N	3.71114	-0.14518	0.00011
C	5.09045	0.34945	0.00026
H	1.27525	-2.33810	-0.00045
H	2.56466	1.68583	0.00038
H	4.07123	-2.27129	-0.00022
H	5.59956	-0.00869	-0.89460
H	5.59964	-0.00954	0.89474
H	5.07204	1.43812	0.00080

Cartesian coordinate of da^{7}G

N	-1.97942	0.24562	-0.00731
N	-2.51996	-2.02234	-0.06737
N	-0.28735	-1.39448	0.01173
C	1.46161	1.76463	0.01120
N	1.92020	-0.43875	-0.00063
C	2.66182	-1.68418	-0.00982
C	-1.54210	-1.05258	-0.00217
C	0.56502	-0.32772	0.00285
C	0.23954	1.03038	0.01189
C	-1.14786	1.39912	0.00175
C	2.46730	0.83509	0.00412
O	-1.66178	2.50398	-0.00911
H	3.29364	-1.75642	0.87888
H	3.28447	-1.74876	-0.90552
H	-2.96797	0.44719	-0.09632
H	-3.38208	-1.83602	0.42563
H	-2.15365	-2.95130	0.08975
H	3.54033	0.95987	0.00183
H	1.94349	-2.50360	-0.00996
H	1.56983	2.83840	0.01480

Table S5. continued.

Cartesian coordinate of da^{7}G-MeIm_1

N	0.24368	1.74103	0.05178
N	1.98878	3.23542	0.19864
N	2.51976	0.99579	0.13671
C	0.64145	-2.04096	-0.23774
N	2.79460	-1.37179	-0.03556
C	4.24783	-1.42782	0.03983
C	1.58443	1.94824	0.12963
C	2.04756	-0.23784	0.02113
C	0.67417	-0.61088	-0.09001
C	-0.16240	0.49565	-0.04216
C	1.94424	-2.44973	-0.19657
N	-1.59469	0.35217	-0.09757
H	4.64976	-1.90163	-0.85747
H	4.55701	-1.98609	0.92546
H	1.30706	3.96744	0.30793
H	2.96574	3.43276	0.34353
H	2.35152	-3.44767	-0.28010
H	4.61714	-0.40527	0.10748
C	-2.46836	1.32852	-0.53027
C	-2.29595	-0.71596	0.29544
C	-3.72091	0.81558	-0.40079
N	-3.58925	-0.45784	0.11773
C	-4.69634	-1.36587	0.43015
H	-2.10424	2.28134	-0.87890
H	-1.88042	-1.61646	0.72189
H	-4.68620	1.23853	-0.63122
H	-5.23988	-1.59842	-0.48576
H	-5.35824	-0.88795	1.15233
H	-4.28959	-2.28118	0.85746
H	-0.20424	-2.69248	-0.39656

Table S5. continued.

Cartesian coordinate of da^{7}G-MeIm_2

N	-0.06074	1.53451	-0.11084
N	1.33438	3.36882	-0.10828
N	2.32104	1.28898	-0.05440
C	1.11268	-2.08765	0.13876
N	3.08215	-0.97003	0.09540
C	4.51571	-0.71620	0.11815
C	1.20639	2.02181	-0.09483
C	2.11634	-0.01952	0.00832
C	0.84828	-0.67900	0.01885
C	-0.19831	0.22607	-0.06486
C	2.47180	-2.20903	0.17895
N	-1.56833	-0.21494	-0.09510
H	4.99303	-1.18233	-0.74582
H	4.94982	-1.10900	1.03934
H	0.52775	3.93987	-0.29790
H	2.25546	3.76233	-0.21715
H	3.07687	-3.10028	0.27031
H	4.66373	0.36222	0.07754
C	-2.04965	-1.43403	-0.52773
C	-2.59912	0.55127	0.27602
C	-3.40283	-1.38797	-0.40119
N	-3.72084	-0.14229	0.10345
C	-5.07526	0.33163	0.40248
H	-1.39889	-2.19982	-0.91648
H	-2.50661	1.56168	0.64497
H	-4.16024	-2.12024	-0.63333
H	-5.51938	-0.30747	1.16569
H	-5.67299	0.30577	-0.50867
H	-5.01382	1.35440	0.77087
H	0.41550	-2.90677	0.22145

Cartesian coordinate of a^{8}da^{7}G

N	-1.97764	0.23998	-0.00421
N	-2.50787	-2.02646	-0.06240
N	-0.28139	-1.40599	0.01014
C	1.48480	1.69042	0.00962
N	1.90990	-0.40692	-0.00089
C	2.73243	-1.59690	-0.00856
C	-1.53747	-1.05934	-0.00117
C	0.56464	-0.33609	0.00224
C	0.23686	1.02191	0.01040
C	-1.14988	1.39769	0.00184
N	2.48071	0.82451	0.00369
O	-1.66335	2.49949	-0.00833
H	3.36567	-1.61116	0.88038
H	3.35834	-1.60487	-0.90274
H	-2.96727	0.43974	-0.08586
H	-3.39998	-1.83357	0.36856
H	-2.15429	-2.96014	0.09208
H	2.06735	-2.46030	-0.00921
H	1.68270	2.75232	0.01299

Table S5. continued.

Cartesian coordinate of a[8]da[7]G-MeIm_1

N	0.24936	1.74638	0.04904
N	1.99442	3.23624	0.19468
N	2.53605	1.00261	0.12888
C	0.70981	-2.02148	-0.21218
N	2.75591	-1.38119	-0.03526
C	4.19887	-1.54188	0.01614
C	1.60275	1.95096	0.12183
C	2.05135	-0.23250	0.02386
C	0.68197	-0.59692	-0.07576
C	-0.16262	0.51063	-0.03499
N	1.94636	-2.45564	-0.18216
N	-1.59300	0.36368	-0.08616
H	4.54671	-2.00201	-0.90936
H	4.46399	-2.17101	0.86646
H	1.30992	3.97236	0.24236
H	2.97565	3.44791	0.28234
H	4.63537	-0.55033	0.13088
C	-2.47615	1.35148	-0.47388
C	-2.28535	-0.72684	0.26350
C	-3.72335	0.82319	-0.36087
N	-3.57957	-0.47079	0.10256
C	-4.67994	-1.39945	0.38142
H	-2.12168	2.32040	-0.78624
H	-1.86376	-1.64353	0.64774
H	-4.69308	1.24776	-0.56913
H	-5.23254	-1.58595	-0.53948
H	-5.33446	-0.96136	1.13485
H	-4.26449	-2.33385	0.75567
H	-0.09233	-2.73254	-0.35172

Table S5. continued.

Cartesian coordinate of a^{8}da^{7}G-MeIm_2

N	-0.06071	1.53869	-0.10147
N	1.32926	3.37266	-0.11085
N	2.33181	1.30511	-0.04991
C	1.18688	-2.05183	0.12211
N	3.05215	-0.97804	0.08847
C	4.49626	-0.82456	0.12123
C	1.21706	2.03060	-0.08714
C	2.12062	-0.00762	0.00538
C	0.85794	-0.66189	0.01161
C	-0.20018	0.23820	-0.06143
N	2.48702	-2.20685	0.16299
N	-1.56554	-0.21199	-0.08847
H	4.93629	-1.34178	-0.73209
H	4.88445	-1.24370	1.05010
H	0.51129	3.94654	-0.22868
H	2.24720	3.78526	-0.16238
H	4.71462	0.24149	0.06861
C	-2.03122	-1.45588	-0.46659
C	-2.60776	0.56016	0.24112
C	-3.38492	-1.41762	-0.35156
N	-3.71918	-0.15260	0.09304
C	-5.08196	0.31889	0.36111
H	-1.37209	-2.23478	-0.81317
H	-2.52869	1.58640	0.56727
H	-4.13312	-2.16817	-0.55382
H	-5.52355	-0.29147	1.14888
H	-5.67152	0.24463	-0.55267
H	-5.03482	1.35772	0.68373
H	0.54995	-2.92180	0.19263
